# Huoxue Qingre decoction used for treatment of coronary heart disease network analysis and metabolomic evaluation

**DOI:** 10.3389/fphar.2022.1025540

**Published:** 2022-10-20

**Authors:** Yu-Qing Tan, Min Jin, Xuan-Hui He, Heng-Wen Chen

**Affiliations:** Department of Pharmacy, Guang’anmen Hospital, China Academy of Chinese Medical Sciences, Beijing, China

**Keywords:** network pharmacology, traditional Chinese medicine (TCM), Huoxue Qingre decoction, coronary heart disease (CHD), metabolomics

## Abstract

**Objective:** Network pharmacology provides new methods and references for the research of traditional Chinese medicine, but some problems remain, such as single evaluation components and index methods, imperfect relevant databases, unscientific prediction results, and lack of verification of results. Herein, we used a modified network pharmacology research method to explore the potential network analysis mechanism of Huoxue Qingre decoction in the treatment of coronary heart disease and utilized clinical trials for assessment.

**Methods:** Based on literature research, the targets corresponding to the drug were obtained with the assistance of the TCMSP database and Swiss Target Prediction, and the target proteins were corrected using the UniProt database. The targets related to coronary heart disease was obtained through the GeneCards database. A protein-protein interaction network diagram was constructed, and a “component-intersection target” network diagram was drawn based on Cytoscape 3.6.2 software. The mapped targets were imported into the DAVID bioinformatics platform, which underwent Gene Ontology (GO) function and Kyoto Encyclopedia of Genes and Genomes (KEGG) pathway enrichment analysis, and the network pharmacology prediction results were evaluated through clinical trials.

**Results:** We obtained 151 compounds related to Huoxue Qingre decoction, 286 genes after evaluation and deduplication, and 426 genes related to coronary heart disease. Finally, 81 common target genes were obtained with 32 pathways according to the KEGG pathway enrichment analysis. The validation results of the clinical trials showed that a total of 98 differential metabolites were found in the treatment of coronary heart disease with Huoxue Qingre decoction, involving a total of 16 metabolic pathways. Compared with the network pharmacology prediction results, it was found that only the pathways in cancer (hsa05200) were the common pathways in the top 32 signaling pathways predicted by network pharmacology. The expanded network pharmacology prediction results revealed that the sphingolipid signaling pathway (hsa04071) and prostate cancer pathway (hsa05215) matched the predicted metabolic pathways, with differential metabolites of N-oleoyl-D-sphingomyelin and 1-methyl-6-phenyl-1h-imidazole[4,5-b]pyridine-2-amine.

**Conclusion:** Through the network analysis and metabolomic evaluation, there may be three signaling pathways that involve the Huoxue Qingre decoction in the treatment of coronary heart disease: pathways in cancer (hsa05200), sphingolipid signaling pathway (hsa04071), and prostate cancer pathway (hsa05215).

## Introduction

Network pharmacology is an emerging, cross and cutting-edge discipline in the systematic study of drugs in the era of artificial intelligence and big data. It emphasizes that the molecular associations between drugs and therapeutic objects can be obtained from the perspective of the system level and the whole of the biological network. It has been widely used in the interpretation of the overall mechanism of action of traditional Chinese medicine (TCM) compounds, and in the analysis of drug combinations and formula compatibility law, providing new ideas for the study of complex systems of TCM (Zhang et al., 2019). In recent years, the number of articles on network pharmacology has rapidly increased every year, with hundreds of articles being published. According to a search with the subject term “network pharmacology,” more than 2,800 Chinese knowledge network studies were conducted in 2021.

Coronary heart disease (CHD), also known as coronary atherosclerotic heart disease, is caused by narrowing or obstruction of the vascular lumen due to atherosclerotic lesions in the coronary vessels, resulting in myocardial ischemia, hypoxia, or necrosis. Coronary atherosclerosis is a factor involved in the pathogenesis of CHD. It has been reported that the current number of patients with cardiovascular diseases in China is 290 million, of which the number of patients with CHD is as high as 11 million, and this has become a major public health problem in China (Benjamin et al., 2017; Hu et al., 2019).

At present, there have been satisfactory results in the use of TCM for the treatment of CHD. The combination of western medicine disease identification and TCM syndrome differentiation has become an important form of clinical diagnosis, treatment, and rehabilitation of CHD. In TCM, blood stasis is considered to be a factor leading to the dystrophy of the heart vessels, which leads to pain when the heart vessels are abnormal, thus causing “chest obstruction” and “heartache.” Its characteristics are basically consistent with the symptoms of modern CHD. Qi is the commander of the blood, and the normal operation of blood depends on the normal promotion of Qi. If the vitality is deficient and unable to circulate the blood, the blood flow will be slow and there will be blood stasis. Therefore, the treatment of CHD should focus on promoting blood circulation and invigorating Qi (China Association of Chinese Medicine Cardiovascular Disease Branch, 2019).

Huoxue Qingre (HXQR) decoction is the clinical experience of Guang’anmen Hospital in the treatment of CHD with significant therapeutic effect. HXQR consists of eight herbs, including *Salvia miltiorrhiza* Bunge (Danshen, DS), *Panax ginseng* C.A.Mey. (Renshen, RS), *Coptis chinensis* Franch. (Huanglian, HL), *Pinellia ternata* (Thunb.) Makino (Fabanxia, BX), *Paeonia lactiflora* Pall. (Chishao, CS), *Panax notoginseng* (Burk) F. H. Chen (Sanqi, SQ), *Pueraria lobata* (Willd.) Maesen & S.M. Almeida ex Sanjappa & Predeep (Gegen, GG), and *Cistanche deserticola* Ma (Roucongrong, RCR). It has the effects of invigorating Qi and activating blood circulation, clearing heat, and removing phlegm. In this formula, *Salvia miltiorrhiza* is the monarch drug. *Panax ginseng*, *Coptis chinensis*, and *Pinellia ternata* are the minister drugs. *Paeonia lactiflora*, *Panax notoginseng*, *Puerariae Lobatae*, and *Cistanche deserticola* are adjuvant drugs. Previously, our group found that HXQR improves the mitochondrial structure and function of cardiomyocytes, inhibits inflammatory factors and myocardial apoptosis, and thus improves ventricular remodeling after myocardial infarction (Chen et al., 2017; Chen et al., 2018; Wang et al., 2019).

Network pharmacology provides new methods and references for TCM research, but problems still remain such as single evaluation components and index methods, imperfect relevant databases, unscientific prediction results, and insufficient validation of results. Therefore, in the current study, we intend to adopt and improve the network pharmacology research method (Zhang and Li, 2015; Xie et al., 2019), explore the potential mechanism of HXQR in the treatment of critical coronary strain, and verify results through clinical trials. This study will provide a demonstration for the in-depth study of network pharmacology, promote the further development and application of network pharmacology, and provide a reference for the clinical application and mechanism research of HXQR decoction and other TCM compounds.

## Materials and methods

### Preparation of HXQR decoction

HXQR was provided by the Department of Pharmacy, Guang’anmen Hospital, China Academy of Chinese Medical Science. It consists of *Salvia miltiorrhiza* Bunge (Danshen, 20.8%, Collection bar code PE02053963, product batch number 15102502), *Panax ginseng* C.A.Mey. (Renshen, 8.3%, Collection bar code PE 01471910, product batch number 15102902), *Coptis chinensis* Franch. (Huanglian, 6.9%, Collection bar code PE01268317, product batch number 15111102), *Pinellia ternata* (Thunb.) Makino (Fabanxia, 8.3%, Collection bar code PE02240636, product batch number SAA171), *Paeonia lactiflora* Pall. (Chishao, 16.7%, Collection bar code PE02247249, product batch number 16011601), *Panax notoginseng* (Burk) F. H. Chen (Sanqi, 4.2%, Collection bar code PE 01976963, product batch number 15041601), *Pueraria lobata* (Willd.) Maesen & S.M. Almeida ex Sanjappa & Predeep (Gegen, 20.8%, Collection bar code PE02015760, product batch number 15121701), and *Cistanche deserticola* Ma (Roucongrong, 13.9%, Collection bar code PE 01542425, product batch number 160260541) (Table 1). All medicinal names have been unified using the Kew Medicinal plant names service. All specimens are deposited in Chinese National Herbarium, Institute of Botany, Chinese Academy of Sciences (20 Nanxincun, Xiangshan, Beijing, China). All medicinal materials are provided by Beijing Kangmei Pharmaceutical Co., Ltd. (Beijing, China).

**TABLE 1:** The composition of traditional Chinese medicine (TCM) in HXQR.

Composed of TCM	Composed of TCM (Chinese)	Medicinal parts	Percentage (%)	Active ingredient content of TCM (%)
*Salvia miltiorrhiza Bunge*	Danshen	Dry roots and rhizomes	20.8	tanshinone IIA 0.25, salvianolic acid B 5.3
*Panax ginseng* C.A.Mey	Renshen	Dry roots and rhizomes	8.3	ginsenoside Rg1 and Re 0.48, ginsenoside Rb1 0.28
*Coptis chinensis* Franch	Huanglian	Dry roots and rhizomes	6.9	berberine 7.6, epiberberine 1.30, coptisine 2.1, palmatine 2.1
*Pinellia ternata* (Thunb.) Makino	Fabanxia	Dry tuber	8.3	—
*Paeonia lactiflora* Pall	Chishao	Dry roots and rhizomes	16.7	Paeoniflorin 3.3
*Panax notoginseng* (Burkill) F.H.Chen	Sanqi	Dry roots and rhizomes	17.4	ginsenoside Rg1, ginsenoside Rb1, and notoginsenoside R1 6.2
*Pueraria lobata* (Willd.) Maesen & S.M. Almeida ex Sanjappa & Predeep	Gegen	Dry tuber	20.8	Puerarin 3.1
*Cistanche deserticola* Ma	Roucongrong	Dry fleshy stem	13.9	Echinacoside and acteoside 0.43

The medicinal herbs of HXQR were extracted by 10 times of water through heating reflux for twice, each time for 1.0 h. The extracts were mixed and concentrated under reduced pressure. The relative density was about 1.12 (measured at 20°C). Ethanol was added to bring the ethanol content to 70%. After 24 h, the extracts were concentrated (density 1.20 at 50°C), and dried in vacuum at 60°C. Finally, all the extracts were crushed and mixed to get an extract of HXQR.

The dry extract yielding rate of HXQR was 26.00%. The contents of active ingredients in HXQR extract were 3.58% for salvianolic acid B, 0.76% for *Coptis chinensis* total alkaloids (berberine, epiberberine, coptisine and palmatine), 0.33% for ginsenoside Rb1, 1.60% for paeoniflorin, 1.95% for puerarin, 0.24% for echinacoside (Chen et al., 2016). All components are tested by HPLC (Aglient 1100, Agilent Technologies, Inc., CA, United States).

### Prediction of the main active ingredients and targets of HXQR decoction

Based on the systematic database of TCM pharmacology and the analysis platform (Traditional Chinese Medicine Systems Pharmacology Database (TCMSP)), the components of the main chemicals in HXQR decoction were retrieved. *S. miltiorrhiza*, *P. ginseng*, *C. chinensis*, *P. ternata*, *P. veitchii*, *P. notoginseng*, *P. lobata*, and *C. deserticola* were searched to establish a chemical database for HXQR with oral bioavailability (OB) ≥30% and drug-like (DL) ≥0.18, combined with the TCM pharmacopoeia and published literature to supplement active ingredients. According to the TCMSP database and Swiss Target Prediction, the targets corresponding to the drug were queried, and the obtained target proteins were evaluated and corrected using the UniProt database, converted into the corresponding standard gene names, and saved in the drug target database for HXQR.

### Obtaining CHD targets

In the GeneCards database, with “coronary heart disease” as the keyword, a search was performed for CHD-related genes. These genes were exported to the Excel program, and gene evaluation was performed with the median, and the median was taken for four times to evaluate the genes with a relevance score greater than the median, and a CHD target database was then established.

### Construction of a target protein-protein interaction (PPI) network for HXQR

Mapping targets of diseases and drugs were obtained. Cytoscape 3.6.2 software was used to associate each compound with the corresponding targets to construct an “active ingredients-common targets” network. The STRING database was used to select the “homo sapiens” identification, construct the PPI network, analyze its network topological parameters, and find the key targets with node degree as an important parameter.

### GO functional enrichment and KEGG biological pathway enrichment analysis

HXQR and CHD mapping targets were imported into the DAVID bioinformatics platform for Gene Ontology (GO) function and Kyoto Encyclopedia of Genes and Genomes (KEGG) pathway enrichment analysis. The *p* value was used as the condition to analyze the pathway of target regulation biology. The main signaling pathways and biological processes of HXQR exerting pharmacological effects were analyzed to explore the possible mechanism of HXQR in the treatment of CHD.

### Molecular docking of the main active components of HXQR with target proteins

The Uniprot database and RCSB PDB protein structure database (https://www.rcsb.org/) were used to find the PDB ID (Protein Data Bank Identification) of key targets, and obtain protein information, and utilized Pymol software to process molecules and saved them as pdb format. The active ingredient information of the drug corresponding to the target in the TCMSP database was downloaded and saved in mol2 format. Molecular docking was performed using SYBYL-X 2.0 software, and the results were analyzed by the obtained total score.

### Assessment of network pharmacology prediction results by metabolomics in clinical trials

There is a comprehensive understanding of the purpose, methods, benefits, and possible risks of this study, which has been registered in the TCM Clinical Trials Registry (http://www.chictr.org.cn/index.aspx; Chi CTR-IOR-17013189). This trial has been approved by the ethics committee (Guang’anmen Hospital, China Academy of Chinese Medical Sciences, No. 2017-083-KY-01).

The clinical trial of HXQR for CHD was conducted by our group according to the registered trial protocol. Only the serum of patients in the control group and experimental group (9:11) was used for metabolomics analysis in the current study, and the metabolomics analysis method was performed according to the Fujisaka method (Fujisaka et al., 2018). The analytical instrument used for this experiment was a liquid chromatography-mass spectrometry system composed of a Waters ACQUITY UPLC ultra-high performance liquid chromatography-tandem AB Triple TOF 5600 high-resolution mass spectrometer.

Chromatographic conditions: chromatographic column ACQUITY UPLC BEH C18 (100 mm × 2.1 mm, 1.7 μm); column temperature 45°C; mobile phase: (A) water (containing 0.1% formic acid), (B) acetonitrile/methanol (2/3) (v/v) (containing 0.1% formic acid); flow rate: 0.4 ml/min; injection volume: 5 μl; elution conditions: A% (95%–0) gradient elution. Mass spectrometric conditions: electrospray ionization (ESI) ion source, the positive and negative ion scanning modes were used for sample mass spectrometric signal acquisition.

## Results

### Main potential active ingredients in HXQR

The chemical constituents of each drug in HXQR were searched by TCMSP, including 67 chemical constituents related to *S. miltiorrhiza*, 14 chemical constituents related to *C. chinensis*, 25 chemical constituents related to *P. ginseng*, 13 chemical constituents related to *P. ternata*, 27 chemical constituents related to *P. veitchii*, 11 chemical constituents related to *P. notoginseng*, five chemical constituents related to *P. lobata*, and eight chemical constituents related to *C. deserticola*. Combined with the results of the pharmacopoeia and literature analysis, nine components, salvianolic acid B, tanshinone I, ginsenoside Rg1, ginsenoside Re, ginsenoside Rb1, notoginsenoside R1, puerarin, echinacoside, and acteoside, were added to obtain a total of 172 active ingredients, and 151 compounds were obtained after deleting the duplicates.

Among them, MOL002714 is a common component of *P. ternata* and *P. veitchii*; MOL002879, MOL005341, MOL005344, and MOL007476 are common components of *P. ginseng* and *P. notoginseng*; MOL005320 and MOL005384 are common components of *P. ginseng* and *C. deserticola*; MOL000098 is a common component of *C. chinensis*, *P. notoginseng*, and *C. deserticola*; MOL002776 is a common component of *S. miltiorrhiza*, *P. ternata*, and *P. ternata*; MOL000449 is a common component of *P. ginseng*, *P. veitchii*, *P. veitchii*, and *P. notoginseng*; and MOL000358 is a common component of *P. ginseng*, *P. ternata*, *P. veitchii*, *P. notoginseng*, *P. lobata*, and *C. deserticola*. The four components with the highest OB were: MOL007064 przewalskin b, MOL007132 (2R)-3-(3,4-dihydroxyphenyl)-2-[(Z)-3-(3,4-dihydroxyphenyl)acryl]oxy-propionic acid, MOL002907 corchoroside aqt, and MOL005314 celabenzine. The four components with the highest DL were: MOL013352 obacunone, MOL012297 puerarin, MOL008875 echinacoside, and MOL008871 marckine. The results are shown in Table 2.

**TABLE 2:** Basic information of chemical constituents of HXQR.

HXQR ingredients	Molecule name	MOL ID	OB/%	DL
*Salvia miltiorrhiza* Bunge	Cryptotanshinone	MOL007088	52.34	0.4
*Salvia miltiorrhiza* Bunge	tanshinone iia	MOL007154	49.89	0.4
*Salvia miltiorrhiza* Bunge	tanshinone i	MOL007157	29.27	0.36
*Salvia miltiorrhiza* Bunge	salvianolic acid b	MOL007074	3.01	0.41
*Salvia miltiorrhiza* Bunge	przewalskin b	MOL007064	110.32	0.44
*Salvia miltiorrhiza* Bunge	(2R)-3-(3,4-dihydroxyphenyl)-2-[(Z)-3-(3,4-dihydroxyphenyl)acryloyl]oxy-propionic acid	MOL007132	109.38	0.35
*Salvia miltiorrhiza* Bunge	(Z)-3-[2-[(E)-2-(3,4-dihydroxyphenyl)vinyl]-3,4-dihydroxy-phenyl]acrylic acid	MOL007140	88.54	0.26
*Salvia miltiorrhiza* Bunge	(6S)-6-hydroxy-1-methyl-6-methylol-8,9-dihydro-7H-naphtho[8,7-g]benzofuran-10,11-quinone	MOL007150	75.39	0.46
*Salvia miltiorrhiza* Bunge	formyltanshinone	MOL007058	73.44	0.42
*Salvia miltiorrhiza* Bunge	miltionone Ⅱ	MOL007120	71.03	0.44
*Salvia miltiorrhiza* Bunge	epidanshenspiroketallactone	MOL007105	68.27	0.31
*Salvia miltiorrhiza* Bunge	(6S)-6-(hydroxymethyl)-1,6-dimethyl-8,9-dihydro-7H-naphtho[8,7-g]benzofuran-10,11-dione	MOL007155	65.26	0.45
*Salvia miltiorrhiza* Bunge	prolithospermic acid	MOL007130	64.37	0.31
*Salvia miltiorrhiza* Bunge	2-(4-hydroxy-3-methoxyphenyl)-5-(3-hydroxypropyl)-7-methoxy-3-benzofurancarboxaldehyde	MOL007050	62.78	0.4
*Salvia miltiorrhiza* Bunge	Przewaquinone B	MOL007068	62.24	0.41
*Salvia miltiorrhiza* Bunge	digallate	MOL000569	61.85	0.26
*Salvia miltiorrhiza* Bunge	Danshenol B	MOL007081	57.95	0.56
*Salvia miltiorrhiza* Bunge	Danshenol A	MOL007082	56.97	0.52
*Salvia miltiorrhiza* Bunge	przewaquinone c	MOL007069	55.74	0.4
*Salvia miltiorrhiza* Bunge	isocryptotanshi-none	MOL007108	54.98	0.39
*Salvia miltiorrhiza* Bunge	neocryptotanshinone	MOL007125	52.49	0.32
*Salvia miltiorrhiza* Bunge	tanshinaldehyde	MOL007079	52.47	0.45
*Salvia miltiorrhiza* Bunge	danshenspiroketallactone	MOL007094	50.43	0.31
*Salvia miltiorrhiza* Bunge	Isotanshinone II	MOL007111	49.92	0.4
*Salvia miltiorrhiza* Bunge	miltionone Ⅰ	MOL007119	49.68	0.32
*Salvia miltiorrhiza* Bunge	deoxyneocryptotanshinone	MOL007098	49.4	0.29
*Salvia miltiorrhiza* Bunge	(E)-3-[2-(3,4-dihydroxyphenyl)-7-hydroxy-benzofuran-4-yl]acrylic acid	MOL007048	48.24	0.31
*Salvia miltiorrhiza* Bunge	6-o-syringyl-8-o-acetyl shanzhiside methyl ester	MOL007051	46.69	0.71
*Salvia miltiorrhiza* Bunge	tanshinone Ⅵ	MOL007156	45.64	0.3
*Salvia miltiorrhiza* Bunge	salvianolic acid g	MOL007141	45.56	0.61
*Salvia miltiorrhiza* Bunge	isoimperatorin	MOL001942	45.46	0.23
*Salvia miltiorrhiza* Bunge	dihydrotanshinoneⅠ	MOL007101	45.04	0.36
*Salvia miltiorrhiza* Bunge	manool	MOL007115	45.04	0.2
*Salvia miltiorrhiza* Bunge	miltirone Ⅱ	MOL007123	44.95	0.24
*Salvia miltiorrhiza* Bunge	3α-hydroxytanshinoneⅡa	MOL007045	44.93	0.44
*Salvia miltiorrhiza* Bunge	Poriferasterol	MOL001659	43.83	0.76
*Salvia miltiorrhiza* Bunge	Dehydrotanshinone II A	MOL002651	43.76	0.4
*Salvia miltiorrhiza* Bunge	sclareol	MOL007077	43.67	0.21
*Salvia miltiorrhiza* Bunge	salvianolic acid j	MOL007142	43.38	0.72
*Salvia miltiorrhiza* Bunge	Przewaquinone E	MOL007152	42.85	0.45
*Salvia miltiorrhiza* Bunge	Tanshindiol B	MOL007151	42.67	0.45
*Salvia miltiorrhiza* Bunge	(6S,7R)-6,7-dihydroxy-1,6-dimethyl-8,9-dihydro-7H-naphtho[8,7-g]benzofuran-10,11-dione	MOL007070	41.31	0.45
*Salvia miltiorrhiza* Bunge	2-isopropyl-8-methylphenanthrene-3,4-dione	MOL007041	40.86	0.23
*Salvia miltiorrhiza* Bunge	przewaquinone f	MOL007071	40.31	0.46
*Salvia miltiorrhiza* Bunge	microstegiol	MOL007118	39.61	0.28
*Salvia miltiorrhiza* Bunge	α-amyrin	MOL006824	39.51	0.76
*Salvia miltiorrhiza* Bunge	neocryptotanshinone ii	MOL007124	39.46	0.23
*Salvia miltiorrhiza* Bunge	dan-shexinkum d	MOL007093	38.88	0.55
*Salvia miltiorrhiza* Bunge	Miltirone	MOL007122	38.76	0.25
*Salvia miltiorrhiza* Bunge	1,2,5,6-tetrahydrotanshinone	MOL001601	38.75	0.36
*Salvia miltiorrhiza* Bunge	dihydrotanshinlactone	MOL007100	38.68	0.32
*Salvia miltiorrhiza* Bunge	przewalskin a	MOL007063	37.11	0.65
*Salvia miltiorrhiza* Bunge	Methylenetanshinquinone	MOL007061	37.07	0.36
*Salvia miltiorrhiza* Bunge	poriferast-5-en-3beta-ol	MOL001771	36.91	0.75
*Salvia miltiorrhiza* Bunge	miltipolone	MOL007121	36.56	0.37
*Salvia miltiorrhiza* Bunge	luteolin	MOL000006	36.16	0.25
*Salvia miltiorrhiza* Bunge	sugiol	MOL002222	36.11	0.28
*Salvia miltiorrhiza* Bunge	C09092	MOL007107	36.07	0.25
*Salvia miltiorrhiza* Bunge	1-methyl-8,9-dihydro-7H-naphtho[5,6-g]benzofuran-6,10,11-trione	MOL007127	34.72	0.37
*Salvia miltiorrhiza* Bunge	NSC 122421	MOL007149	34.49	0.28
*Salvia miltiorrhiza* Bunge	4-methylenemiltirone	MOL007049	34.35	0.23
*Salvia miltiorrhiza* Bunge	5,6-dihydroxy-7-isopropyl-1,1-dimethyl-2,3-dihydrophenanthren-4-one	MOL007036	33.77	0.29
*Salvia miltiorrhiza* Bunge	salvilenone Ⅰ	MOL007143	32.43	0.23
*Salvia miltiorrhiza* Bunge	3-beta-Hydroxymethyllenetanshiquinone	MOL007059	32.16	0.41
*Salvia miltiorrhiza* Bunge	salviolone	MOL007145	31.72	0.24
*Salvia miltiorrhiza* Bunge	Salvilenone	MOL007085	30.38	0.38
*Coptis chinensis* Franch	palmatine	MOL000785	64.6	0.65
*Coptis chinensis* Franch	berberine	MOL001454	36.86	0.78
*Coptis chinensis* Franch	coptisine	MOL001458	30.67	0.86
*Coptis chinensis* Franch	epiberberine	MOL002897	43.09	0.78
*Coptis chinensis* Franch	Corchoroside A_qt	MOL002907	104.95	0.78
*Coptis chinensis* Franch	Moupinamide	MOL008647	86.71	0.26
*Coptis chinensis* Franch	Magnograndiolide	MOL000622	63.71	0.19
*Coptis chinensis* Franch	(R)-Canadine	MOL002903	55.37	0.77
*Coptis chinensis* Franch	Worenine	MOL002668	45.83	0.87
*Coptis chinensis* Franch	Obacunone	MOL013352	43.29	0.77
*Coptis chinensis* Franch	Berlambine	MOL002904	36.68	0.82
*Coptis chinensis* Franch	berberrubine	MOL002894	35.74	0.73
*Coptis chinensis* Franch	Palmidin A	MOL000762	35.36	0.65
*Panax ginseng* C.A.Mey	Ginsenoside Re	MOL005338	4.27	0.12
*Panax ginseng* C.A.Mey	Celabenzine	MOL005314	101.88	0.49
*Panax ginseng* C.A.Mey	Aposiopolamine	MOL005308	66.65	0.22
*Panax ginseng* C.A.Mey	Frutinone A	MOL005321	65.9	0.34
*Panax ginseng* C.A.Mey	Inermin	MOL003648	65.83	0.54
*Panax ginseng* C.A.Mey	Girinimbin	MOL005356	61.22	0.31
*Panax ginseng* C.A.Mey	Fumarine	MOL000787	59.26	0.83
*Panax ginseng* C.A.Mey	malkangunin	MOL005360	57.71	0.63
*Panax ginseng* C.A.Mey	kaempferol	MOL000422	41.88	0.24
*Panax ginseng* C.A.Mey	Dianthramine	MOL005318	40.45	0.2
*Panax ginseng* C.A.Mey	ginsenoside Rg5_qt	MOL005401	39.56	0.79
*Panax ginseng* C.A.Mey	Deoxyharringtonine	MOL005317	39.27	0.81
*Panax ginseng* C.A.Mey	Chrysanthemaxanthin	MOL004492	38.72	0.58
*Panax ginseng* C.A.Mey	alexandrin_qt	MOL005399	36.91	0.75
*Panax ginseng* C.A.Mey	Panaxadiol	MOL005376	33.09	0.79
*Panax ginseng* C.A.Mey	Gomisin B	MOL005357	31.99	0.83
*Panax ginseng* C.A.Mey	Ginsenoside-Rh4_qt	MOL005348	31.11	0.78
*Pinellia ternata* (Thunb.) Makino	(3S,6S)-3-(benzyl)-6-(4-hydroxybenzyl)piperazine-2,5-quinone	MOL006957	46.89	0.27
*Pinellia ternata* (Thunb.) Makino	beta-D-Ribofuranoside, xanthine-9	MOL006967	44.72	0.21
*Pinellia ternata* (Thunb.) Makino	12,13-epoxy-9-hydroxynonadeca-7,10-dienoic acid	MOL006937	42.15	0.24
*Pinellia ternata* (Thunb.) Makino	10,13-eicosadienoic	MOL006936	39.99	0.2
*Pinellia ternata* (Thunb.) Makino	Cycloartenol	MOL003578	38.69	0.78
*Pinellia ternata* (Thunb.) Makino	24-Ethylcholest-4-en-3-one	MOL001755	36.08	0.76
*Pinellia ternata* (Thunb.) Makino	Cavidine	MOL002670	35.64	0.81
*Pinellia ternata* (Thunb.) Makino	coniferin	MOL000519	31.11	0.32
*Pinellia ternata* (Thunb.) Makino	gondoic acid	MOL005030	30.7	0.2
*Paeonia lactiflora* Pall	paeoniflorin	MOL001924	53.87	0.79
*Paeonia lactiflora* Pall	paeoniflorgenone	MOL001918	87.59	0.37
*Paeonia lactiflora* Pall	paeoniflorin_qt	MOL001925	68.18	0.4
*Paeonia lactiflora* Pall	1-o-beta-d-glucopyranosylpaeonisuffrone_qt	MOL006996	65.08	0.35
*Paeonia lactiflora* Pall	evofolinB	MOL007022	64.74	0.22
*Paeonia lactiflora* Pall	9-ethyl-neo-paeoniaflorin A_qt	MOL007018	64.42	0.3
*Paeonia lactiflora* Pall	(2R,3R)-4-methoxyl-distylin	MOL006992	59.98	0.3
*Paeonia lactiflora* Pall	4-ethyl-paeoniflorin_qt	MOL007008	56.87	0.44
*Paeonia lactiflora* Pall	4-o-methyl-paeoniflorin_qt	MOL007012	56.7	0.43
*Paeonia lactiflora* Pall	(+)-catechin	MOL000492	54.83	0.24
*Paeonia lactiflora* Pall	Lactiflorin	MOL001921	49.12	0.8
*Paeonia lactiflora* Pall	Albiflorin_qt	MOL007005	48.7	0.33
*Paeonia lactiflora* Pall	ellagic acid	MOL001002	43.06	0.43
*Paeonia lactiflora* Pall	Spinasterol	MOL004355	42.98	0.76
*Paeonia lactiflora* Pall	campest-5-en-3beta-ol	MOL005043	37.58	0.71
*Paeonia lactiflora* Pall	stigmast-7-en-3-ol	MOL006999	37.42	0.75
*Paeonia lactiflora* Pall	1-o-beta-d-glucopyranosyl-8-o-benzoylpaeonisuffrone_qt	MOL006994	36.01	0.3
*Paeonia lactiflora* Pall	Ethyl oleate (NF)	MOL002883	32.4	0.19
*Paeonia lactiflora* Pall	8-debenzoylpaeonidanin	MOL007014	31.74	0.45
*Paeonia lactiflora* Pall	benzoyl paeoniflorin	MOL007003	31.14	0.54
*Paeonia lactiflora* Pall	isobenzoylpaeoniflorin	MOL007025	31.14	0.54
*Paeonia lactiflora* Pall	(1S,2S,4R)-trans-2-hydroxy-1,8-cineole-B-D-glucopyranoside	MOL006990	30.25	0.27
*Paeonia lactiflora* Pall	Albiflorin	MOL007004	30.25	0.77
*Panax notoginseng* (Burk) F. H. Chen	notoginsenoside r1	MOL007487	5.43	0.13
*Panax notoginseng* (Burk) F. H. Chen	Mandenol	MOL001494	42	0.19
*Panax notoginseng* (Burk) F. H. Chen	ginsenoside f2	MOL007475	36.43	0.25
*Panax notoginseng* (Burk) F. H. Chen	DFV	MOL001792	32.76	0.18
*Pueraria lobata* (Willd.) Maesen & S.M. Almeida ex Sanjappa & Predeep	puerarin	MOL012297	24.03	0.69
*Pueraria lobata* (Willd.) Maesen & S.M. Almeida ex Sanjappa & Predeep	formononetin	MOL000392	69.67	0.21
*Pueraria lobata* (Willd.) Maesen & S.M. Almeida ex Sanjappa & Predeep	3′-Methoxydaidzein	MOL002959	48.57	0.24
*Pueraria lobata* (Willd.) Maesen & S.M. Almeida ex Sanjappa & Predeep	Daidzein-4,7-diglucoside	MOL003629	47.27	0.67
*Cistanche deserticola* Ma	acteoside	MOL003333	2.94	0.62
*Cistanche deserticola* Ma	echinacoside	MOL008875	3.14	0.38
*Cistanche deserticola* Ma	Yangambin	MOL007563	57.53	0.81
*Cistanche deserticola* Ma	Marckine	MOL008871	37.05	0.69
*Pinellia ternata* (Thunb.) Makino, *Paeonia lactiflora* Pall	baicalein	MOL002714	33.52	0.21
*Panax ginseng* C.A.Mey., *Panax notoginseng* (Burk) F. H. Chen	Diop	MOL002879	43.59	0.39
*Panax ginseng* C.A.Mey., *Panax notoginseng* (Burk) F. H. Chen	Sanchinoside C1	MOL005341	10.04	0.28
*Panax ginseng* C.A.Mey., *Panax notoginseng* (Burk) F. H. Chen	ginsenoside rh2	MOL005344	36.32	0.56
*Panax ginseng* C.A.Mey., *Panax notoginseng* (Burk) F. H. Chen	ginsenoside Rb1	MOL007476	6.24	0.04
*Panax ginseng* C.A.Mey., *Cistanche deserticola* Ma	arachidonate	MOL005320	45.57	0.2
*Panax ginseng* C.A.Mey., *Cistanche deserticola* Ma	suchilactone	MOL005384	57.52	0.56
*Coptis chinensis* Franch, *Panax notoginseng* (Burk) F. H. Chen, *Cistanche deserticola* Ma	quercetin	MOL000098	46.43	0.28
*Salvia miltiorrhiza* Bunge, *Pinellia ternata* (Thunb.) Makino, *Paeonia lactiflora* Pall	Baicalin	MOL002776	40.12	0.75
*Panax ginseng* C.A.Mey., *Pinellia ternata* (Thunb.) Makino, *Paeonia lactiflora* Pall., *Panax notoginseng* (Burk) F. H. Chen	Stigmasterol	MOL000449	43.83	0.76
*Panax ginseng* C.A.Mey., *Pinellia ternata* (Thunb.) Makino, *Paeonia lactiflora* Pall., *Panax notoginseng* (Burk) F. H. Chen, *Pueraria lobata* (Willd.) Maesen & S.M. Almeida ex Sanjappa & Predeep, *Cistanche deserticola* Ma	beta-sitosterol	MOL000358	36.91	0.75

### Main active ingredient-target network for HXQR

**FIGURE 1:**
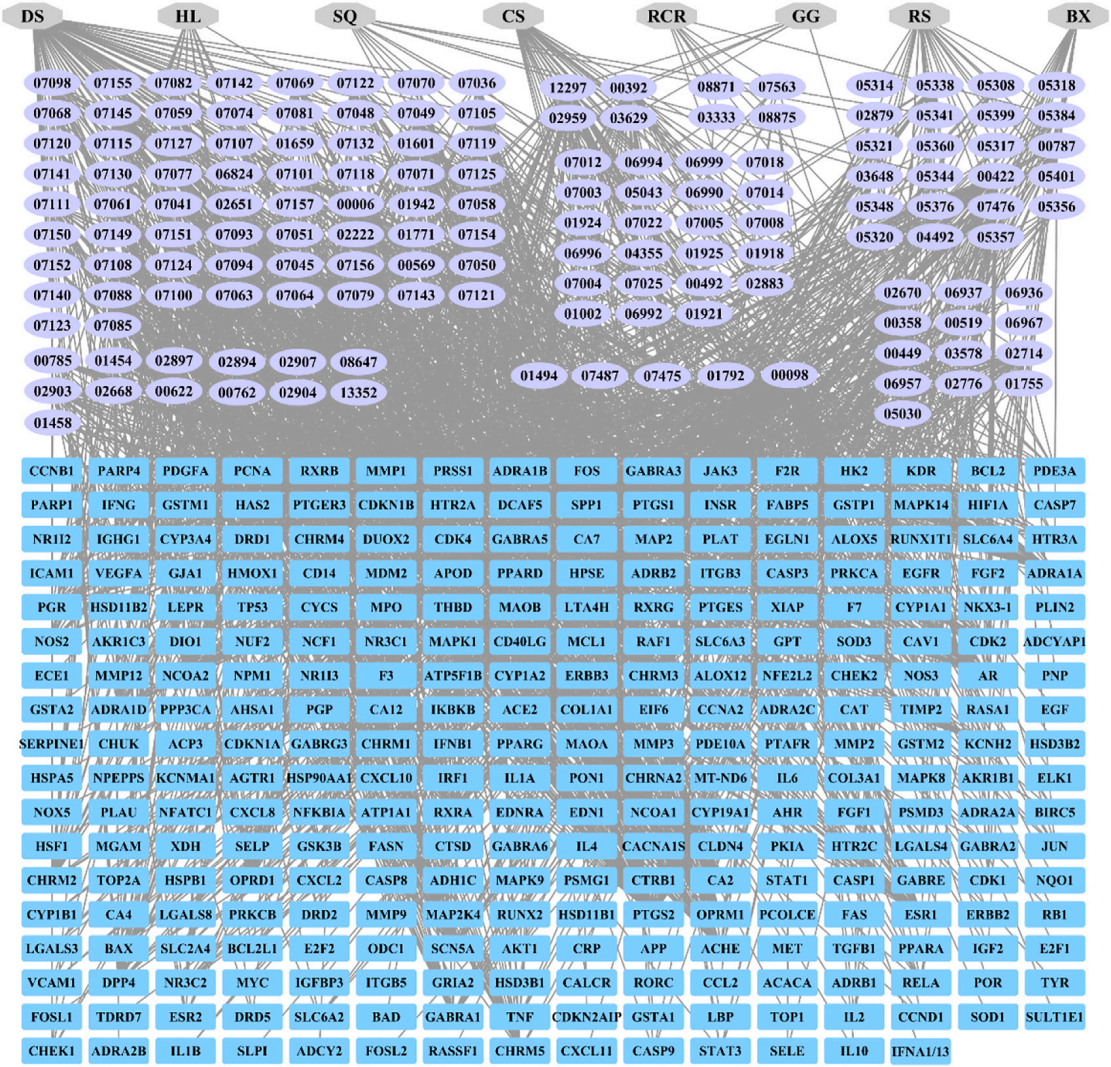
HXQR-active ingredients-drug targets.

A total of 2166 constituent targets were retrieved through the TCMSP database and the UniProt website (https://www.uniprot.org/). Human genes corresponding to TCM drug targets were searched to remove duplicates, and finally, 286 genes were obtained. A network diagram of “HXQRs-active ingredient-drug targets” was drawn. The active ingredients in the figure are expressed according to the last five digits of the MOD ID. TCM drugs are represented by initials. There are a total of 445 nodes and 1734 edges, as shown in Figure 1.

### Prediction of potential targets of HXQR in the treatment of CHD

**FIGURE 2:**
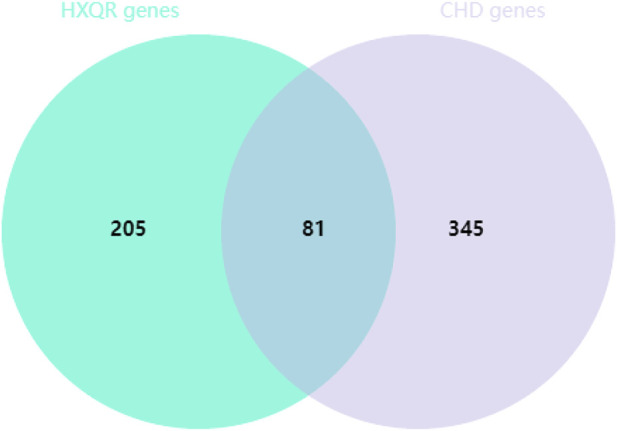
Intersection of active ingredients of HXQR and CHD targets.

A search was performed in the GeneCards database to find the median genes related to coronary heart disease, and the genes with a relevance score greater than the median were further analyzed. Subsequently, 426 targets were obtained that intersected with the targets of the main active components of HXQR, which yielded 81 common target genes, as shown in Figure 2. Cytoscape 3.7.2 was used to visualize the relationship between the targets of the active ingredients of HXQR in the treatment of CHD. The analysis showed that HXQR involved 110 active components in the treatment of CHD, with 190 nodes and 523 edges in the network diagram. The node degree reflects the importance of nodes in the network. The area of the network diagram was directly proportional to the area ratio using the degree reaction.

**FIGURE 3:**
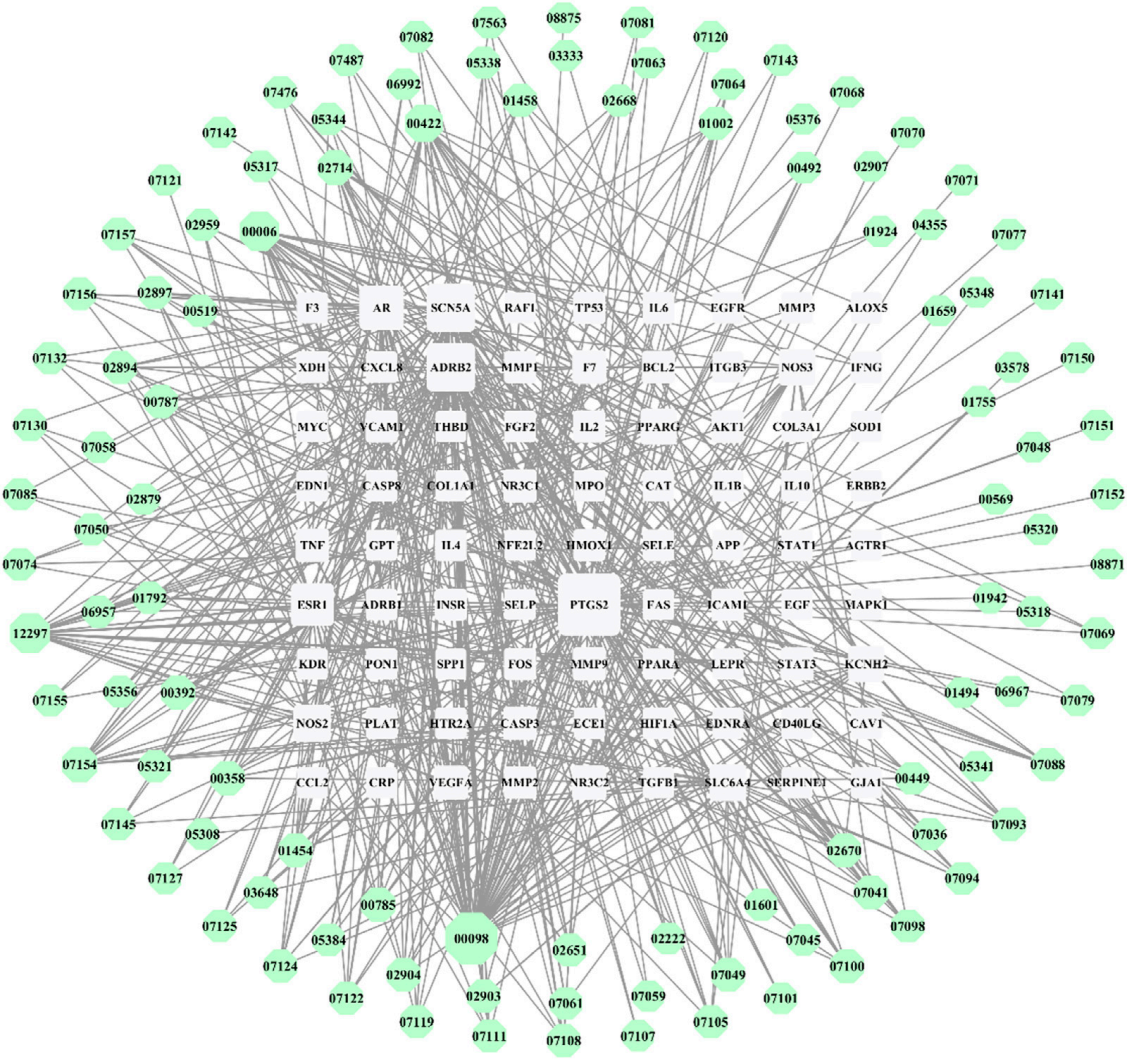
Active ingredients–targets of HXQR.

The results showed that prostaglandin G/H synthase 2 (PTGS2), β-2 adrenergic receptor (ADRB2), sodium channel protein type 5 subunit α (SCN5A), androgen receptor (AR), estrogen receptor (ESR1), inducible nitric oxide lyase (NOS2), sodium-dependent serotonin transporter (SLC6A4), endothelial nitric oxide synthase (NOS3), potassium voltage-gated channel subfamily H member 2 (KCNH2), peroxisome proliferator-activated receptor γ (PPARG), and other targets were the core nodes of the network, as shown in Figure 3. In terms of compounds, quercetin (MOL000098), puerarin (MOL012297), luteolin (MOL000006), kaempferol (MOL000422), tanshinone iia (MOL007154), and baicalein (MOL002714) were associated with multiple targets and may be the main active components of HXQR decoction in the treatment of CHD.

### HXQR-targets-CHD PPI network construction

**FIGURE 4:**
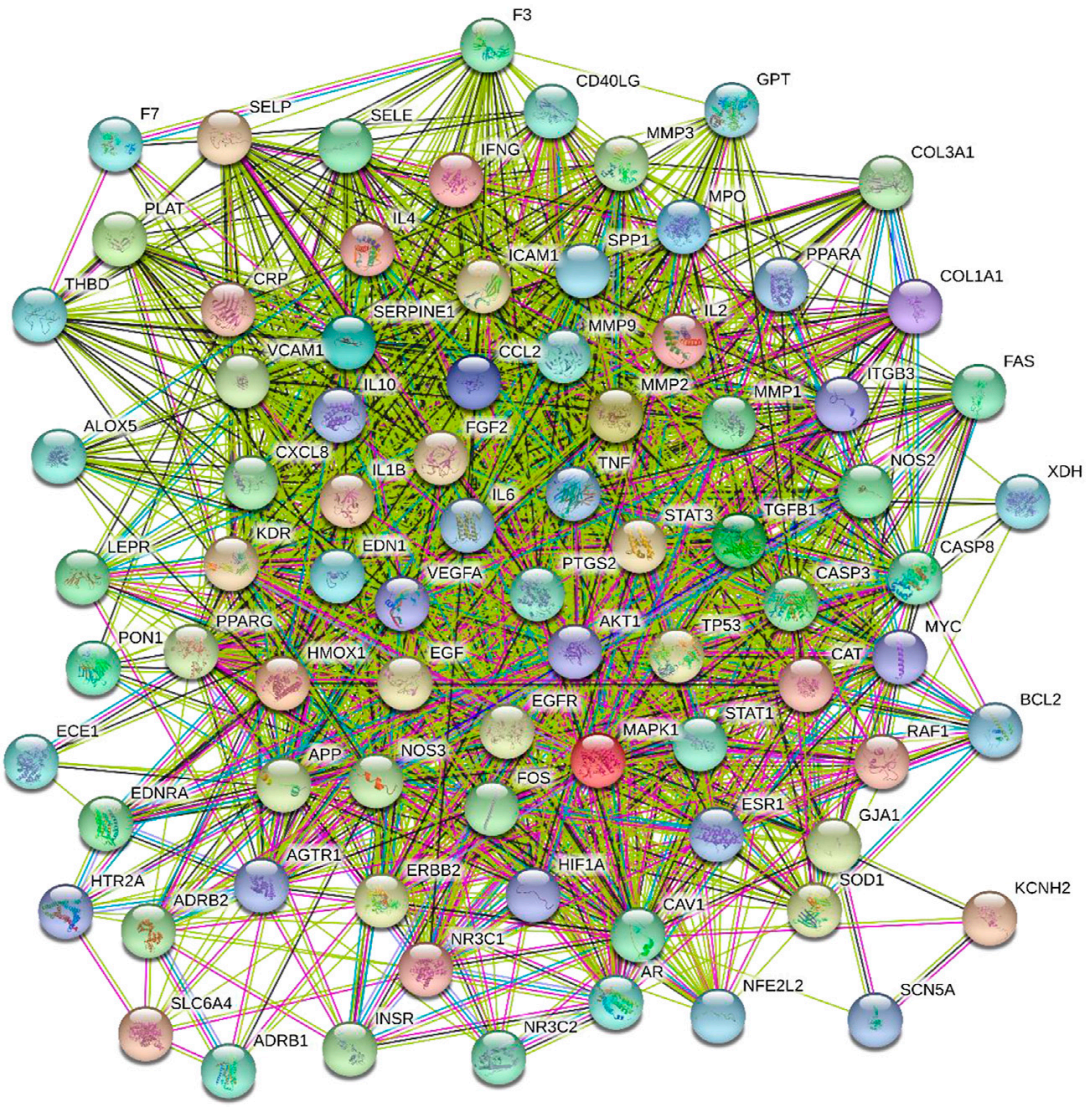
PPI network diagram of the targets of CHD treated by HXQR.

The drug and disease intersection targets from 81 mappings were analyzed by the STRING database to construct the potential target PPI network, and the results were shown in Figure 4. There were 81 targets in the PPI network that had the ability to interact with proteins, and 1514 edges represented the interactions between proteins. The average degree value was 37.383. There were 45 target proteins with a degree value greater than that of the average. The top 20 were IL6, AKT1, VEGFA, TNF, CCL2, IL1B, EGF, CASP3, CXCL8, MAPK1, MMP9, EDN1, PTGS2, NOS3, SERPINE1, IL10, TP53, EGFR, ICAM1, and MMP2, which suggested that these proteins might play a key role in the treatment of CHD with HXQR.

### GO functional enrichment of HXQR

**FIGURE 5:**
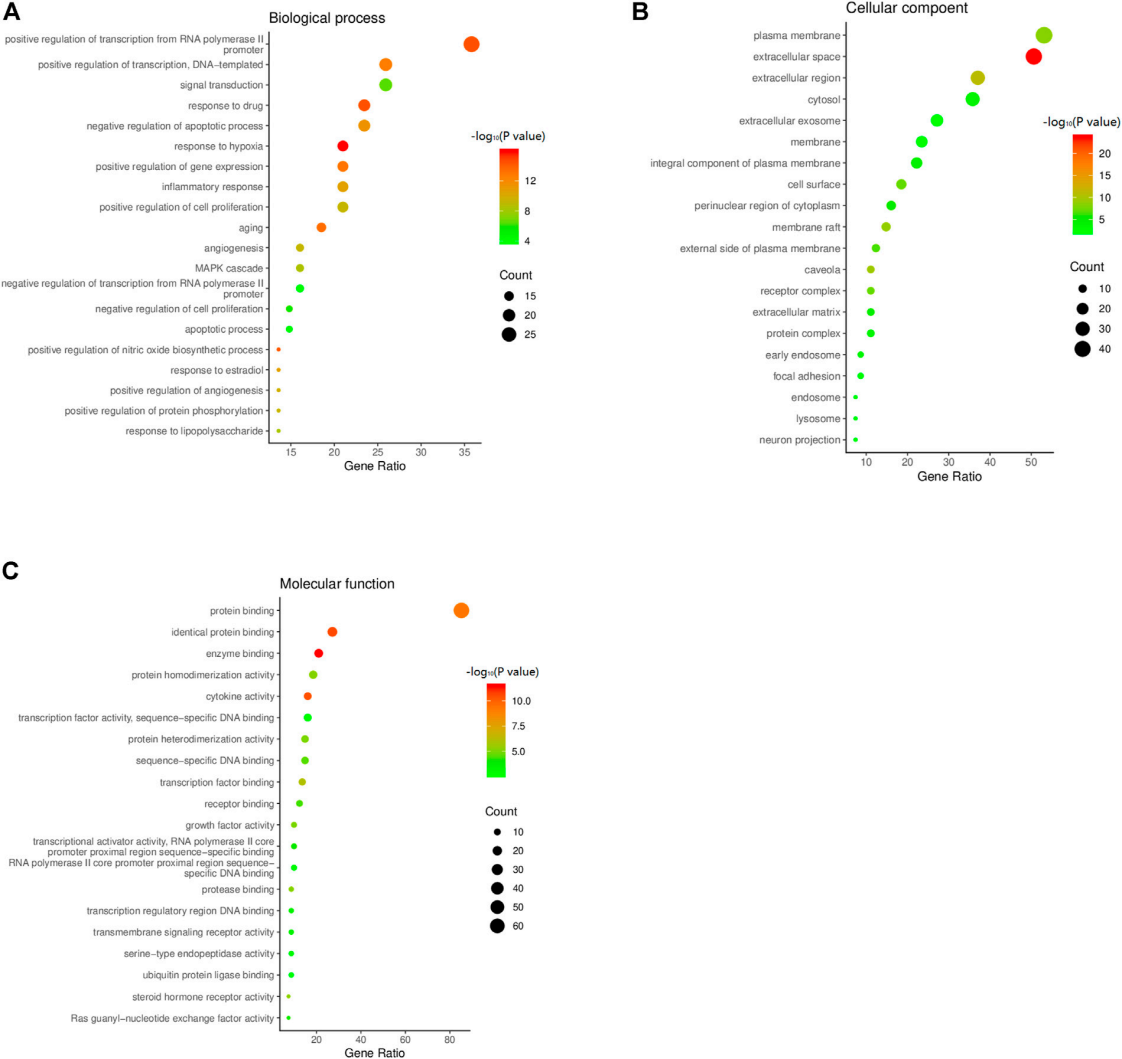
**(A)** Biological process of GO function bubble chart of the targets of CHD treated by HXQR. **(B)** Cellular compoent of GO function bubble chart of the targets of CHD treated by HXQR. **(C)** Molecular function of GO function bubble chart of the targets of CHD treated by HXQR.

The common targets of HXQR and CHD were subjected to GO enrichment analysis and included three aspects: biological process (BP), cellular component (CC), and molecular function (MF), and at *p* < 0.01, 254 items were obtained for BP, 26 items for CC, and 41 items for MF. BPs mainly involve positive regulation of transcription by the RNA polymerase II promoter, positive regulation of transcription, DNA template, gene expression, signal transduction, inflammatory response, cell proliferation, aging, drug response, negative regulation of apoptotic process, and response to hypoxia; CCs mainly involve the plasma membrane, extracellular space, extracellular region, cytosol, and extracellular exosome; MFs mainly involve protein binding, identical protein binding, enzyme binding, protein homodimerization activity, and cytokine activity. Among them, three aspects are in the descending order of enrichment number, and the first 20 items were plotted. The *p* value is taken as the negative logarithm in the figure, and the significance is expressed by the depth of color. The number of enriched genes is expressed by the size of the bubbles, and the greater the number, the larger the bubble, as shown in Figures 5A–C.

### KEGG pathway enrichment analysis

**FIGURE 6:**
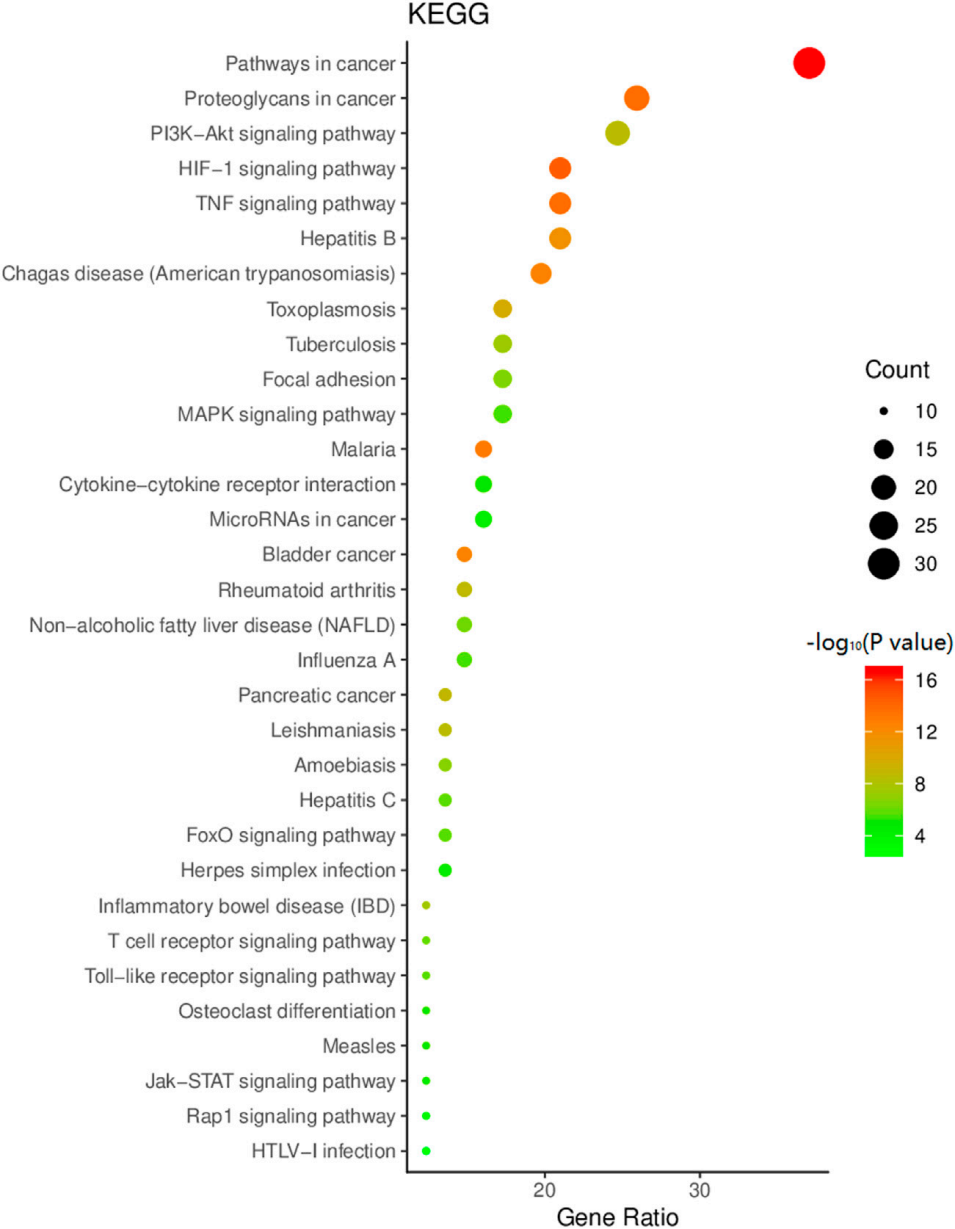
KEGG enrichment analysis bubble diagram.

KEGG pathway enrichment analysis was performed on the common targets of HXQR and CHD, and it was determined that 105 pathways were associated with them. Evaluating with *p* < 0.05 and number of genes greater than or equal to 10 resulted in 32 pathways. Among them, the enrichment number is arranged in descending order, and the bubble is plotted as shown in Figure 6, in which the *p* value is taken as the negative logarithm, the color indicates the size, and the gene number is expressed as the bubble size.

### Molecular docking analysis

**FIGURE 7:**
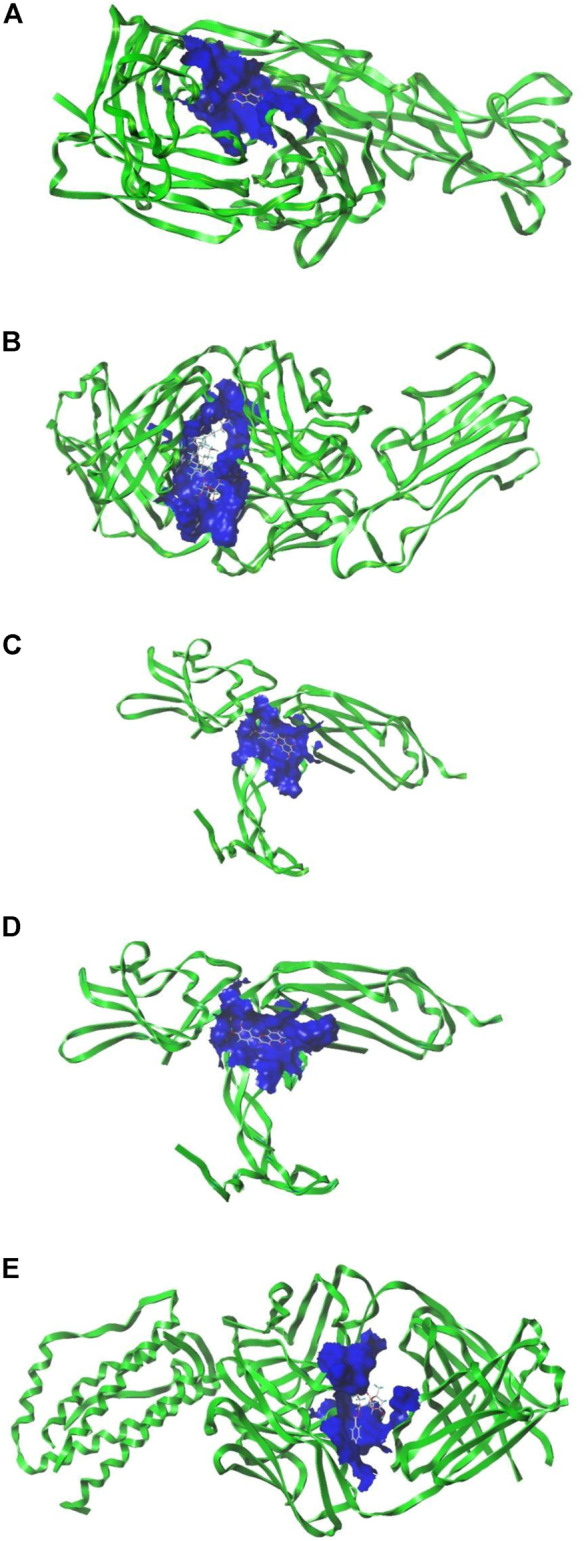
**(A)** Docking diagram of TNF- kaempferol. **(B)** Docking diagram of TNF- ginsenoside rh2. **(C)** Docking diagram of VEGFA-luteolin. **(D)** Docking diagram of VEGFA-quercetin. **(E)** Docking diagram of IL6-paeoniflorin.

Five key targets with high relevance in the PPI network: IL6, AKT1, VEGFA, TNF, and CCL2 were selected to obtain PDB IDs and target structures, and the five targets were docked to their corresponding active ingredients, respectively, and when the target was combined with the compound structure, the higher the docking score, the more stable the binding of the target with the chemical (Table 3). The average docking score was 4.9 points, which showed good binding strength, and 12 of them ≥5 points, suggesting that the core target has good binding ability with HXQR decoction active ingredients. Most of the compounds were linked to the core target in hydrogen-hydrogen bonds and arranged in descending order of docking score, and the docking structure diagram with a docking score greater than six is shown below (Figures 7A–E).

**TABLE 3:** Docking scores between key targets and drug components.

Target	MOL ID		PDB ID	Total score
IL6	MOL000006	luteolin	4o9h	5.89
IL6	MOL000098	quercetin	4o9h	4.57
IL6	MOL001924	paeoniflorin	4o9h	6.13
AKT1	MOL000006	luteolin	3o96	5.24
AKT1	MOL000098	quercetin	3o96	5.27
AKT1	MOL000422	kaempferol	3o96	4.4
AKT1	MOL002714	baicalein	3o96	3.97
AKT1	MOL012297	puerarin	3o96	5.86
VEGFA	MOL000006	luteolin	3v2a	6.37
VEGFA	MOL000098	quercetin	3v2a	6.2
VEGFA	MOL001002	ellagic acid	3v2a	4.41
VEGFA	MOL002714	baicalein	3v2a	4.5
VEGFA	MOL005338	Ginsenoside Re	3v2a	5.48
VEGFA	MOL007157	tanshinone i	3v2a	3.45
VEGFA	MOL007476	ginsenoside Rb1	3v2a	4.48
VEGFA	MOL007487	notoginsenoside r1	3v2a	2.5
VEGFA	MOL012297	puerarin	3v2a	5.64
TNF	MOL000006	luteolin	3wd5	4.46
TNF	MOL000098	quercetin	3wd5	4.07
TNF	MOL000422	kaempferol	3wd5	6.47
TNF	MOL001924	paeoniflorin	3wd5	5.7
TNF	MOL005344	ginsenoside rh2	3wd5	6.44
TNF	MOL007088	cryptotanshinone	3wd5	2.57
TNF	MOL012297	puerarin	3wd5	3.94
CCL2	MOL000098	quercetin	1dok	4.63

### Analysis of metabolomics assessment results in clinical trials

**FIGURE 8:**
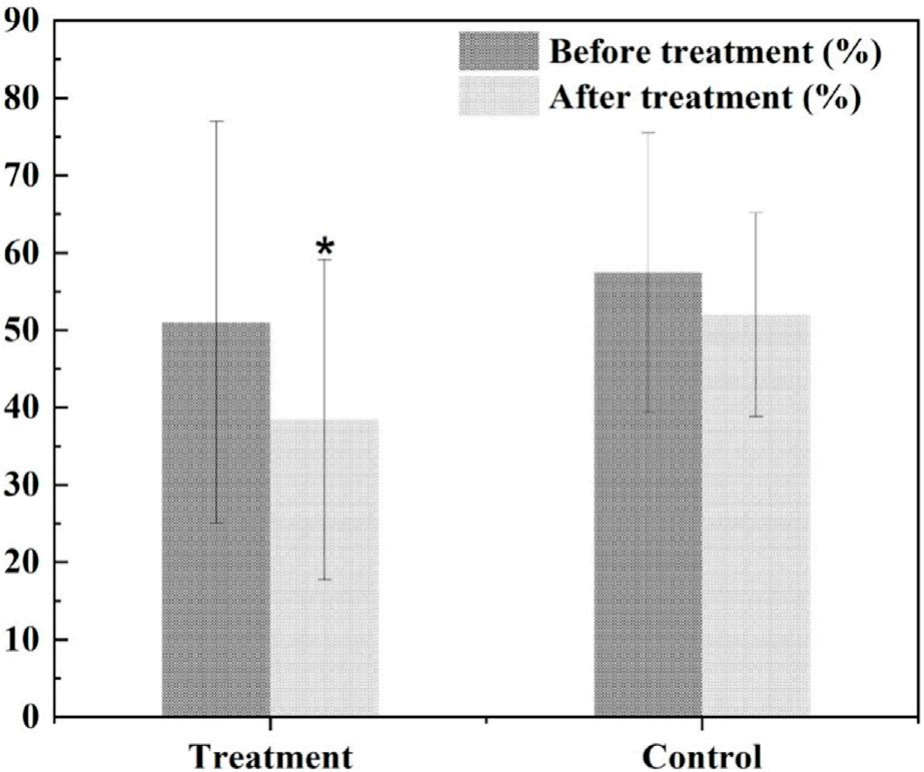
The degree of stenosis of the target plaque area. *p < 0.05 compared with the control.

The research group carried out a small sample randomized double-blind study in the early stage. 32 patients with borderline coronary artery disease were included and randomly divided into the experimental group and the control group according to the ratio of 1:1. Both groups received conventional western medicine treatment. On this basis, the test group was given HXQR, while the control group was given HXQR placebo. After 6 months of treatment, the study found that compared with the control group, the degree of stenosis of the target plaque area in the experimental group was significantly reduced after treatment (*p* = 0.033) (Figure 8).

**FIGURE 9:**
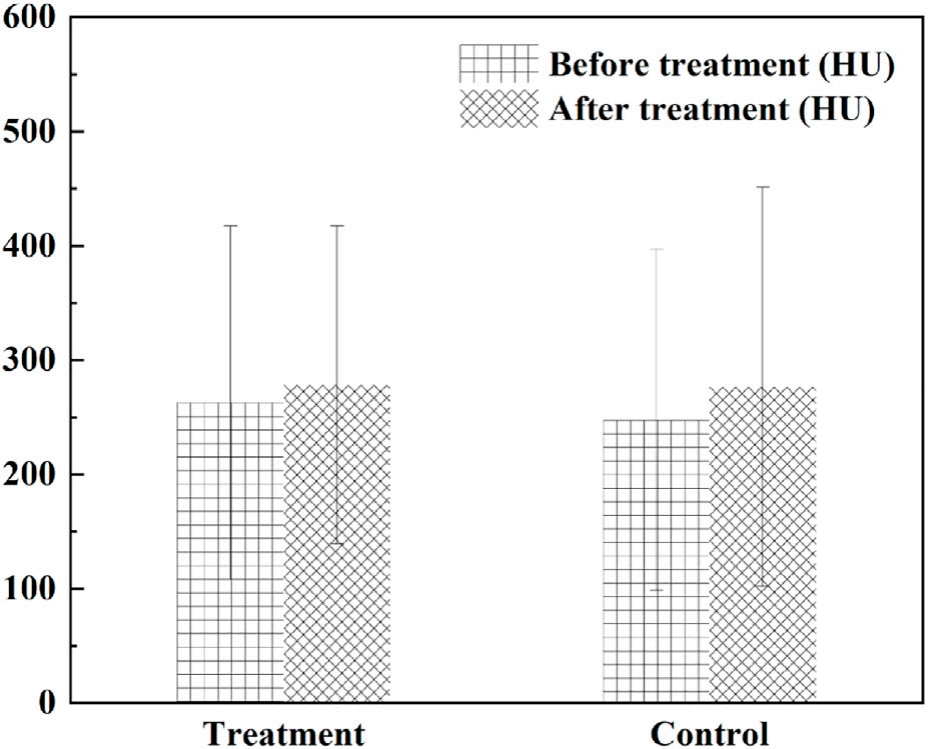
The average CT value of the target plaque.

**FIGURE 10:**
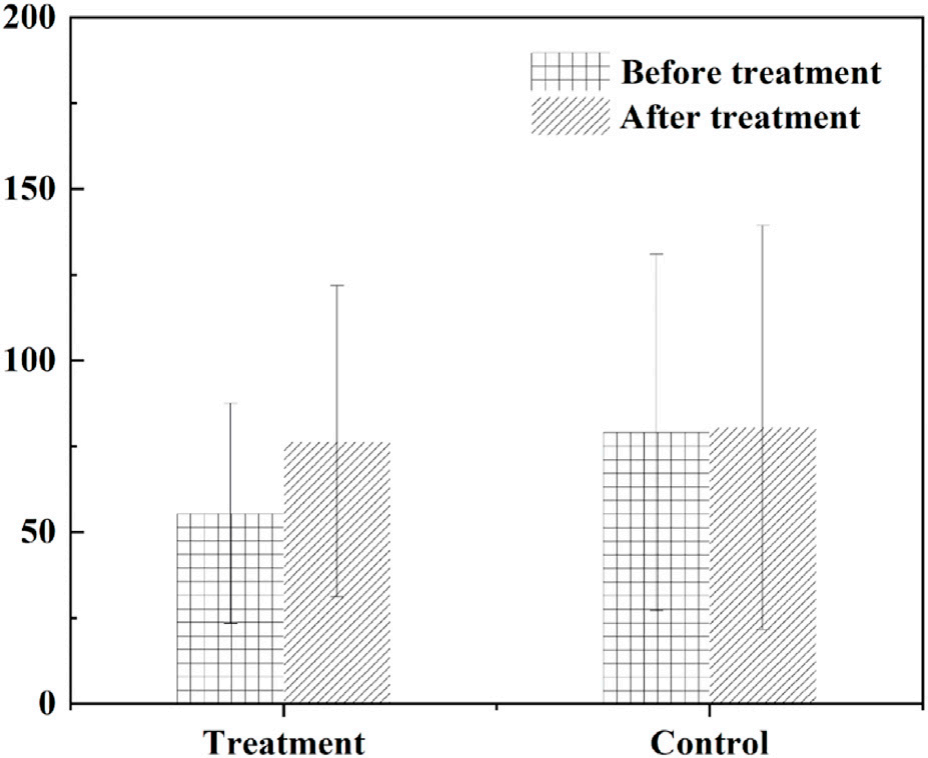
The calcification score of the target plaque.

The ratios were (38.42 ± 20.68)% and (52.00 ± 13.15)%, respectively, while there was no statistical difference between the average CT value of the target plaque and the calcification score of the target plaque (Figures 9, 10).

**FIGURE 11:**
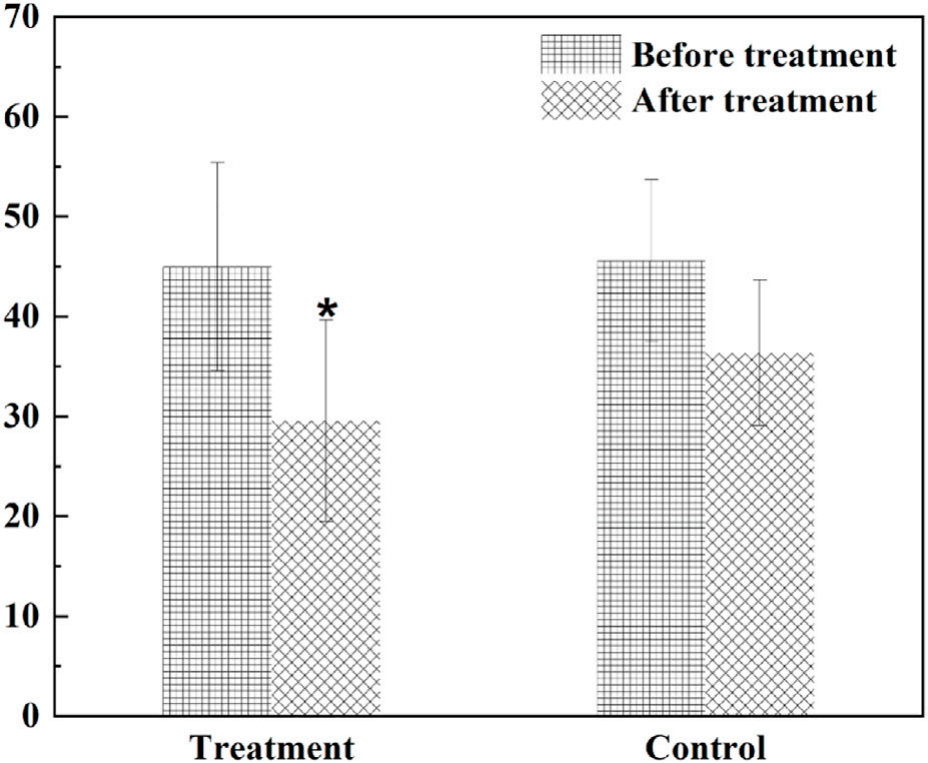
The TCM symptom score of angina pectoris of CHD. *p < 0.05 compared with the control.

The TCM symptom score of angina pectoris of CHD showed a significantly higher decrease of the treatment score than that of the control group (*p* = 0.028) (Figure 11), and the symptoms of chest tightness and palpitations were significantly improved. Before and after the treatment, there was no significant difference in total cholesterol, triglyceride, high density lipoprotein, low density lipoprotein and very low-density lipoprotein between the two groups. The blood and urine routine, liver and kidney function and electrocardiogram were within the normal range before and after treatment in the two groups, with no significant difference. The study showed that HXQR for patients with borderline coronary artery disease could significantly improve chest tightness and palpitations, and had a good effect in stabilizing plaques and improving the degree of coronary stenosis (Huang, 2019).

**FIGURE 12:**
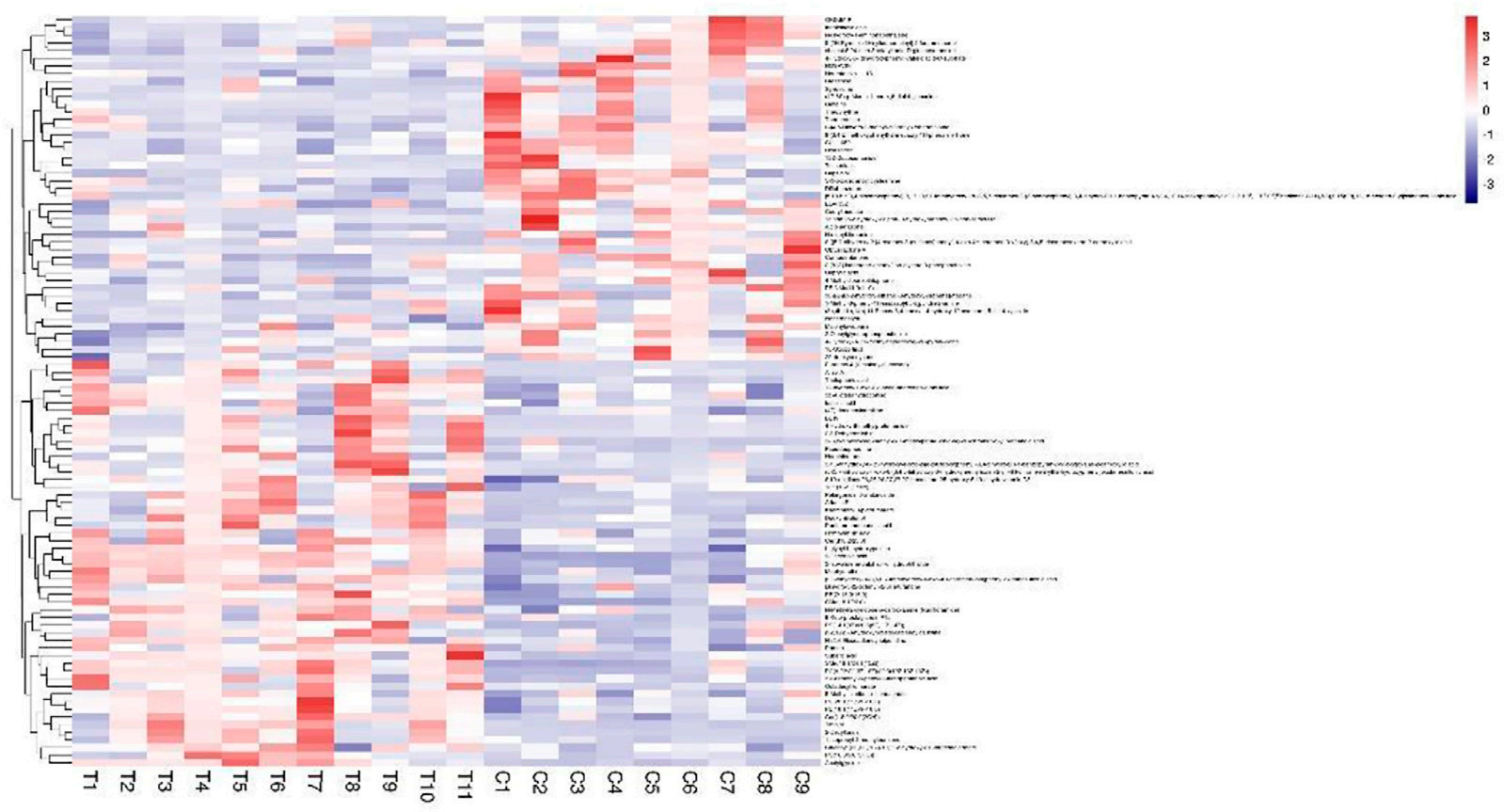
Hotspot chart of metabolomics differential metabolites in clinical trials.

**FIGURE 13:**
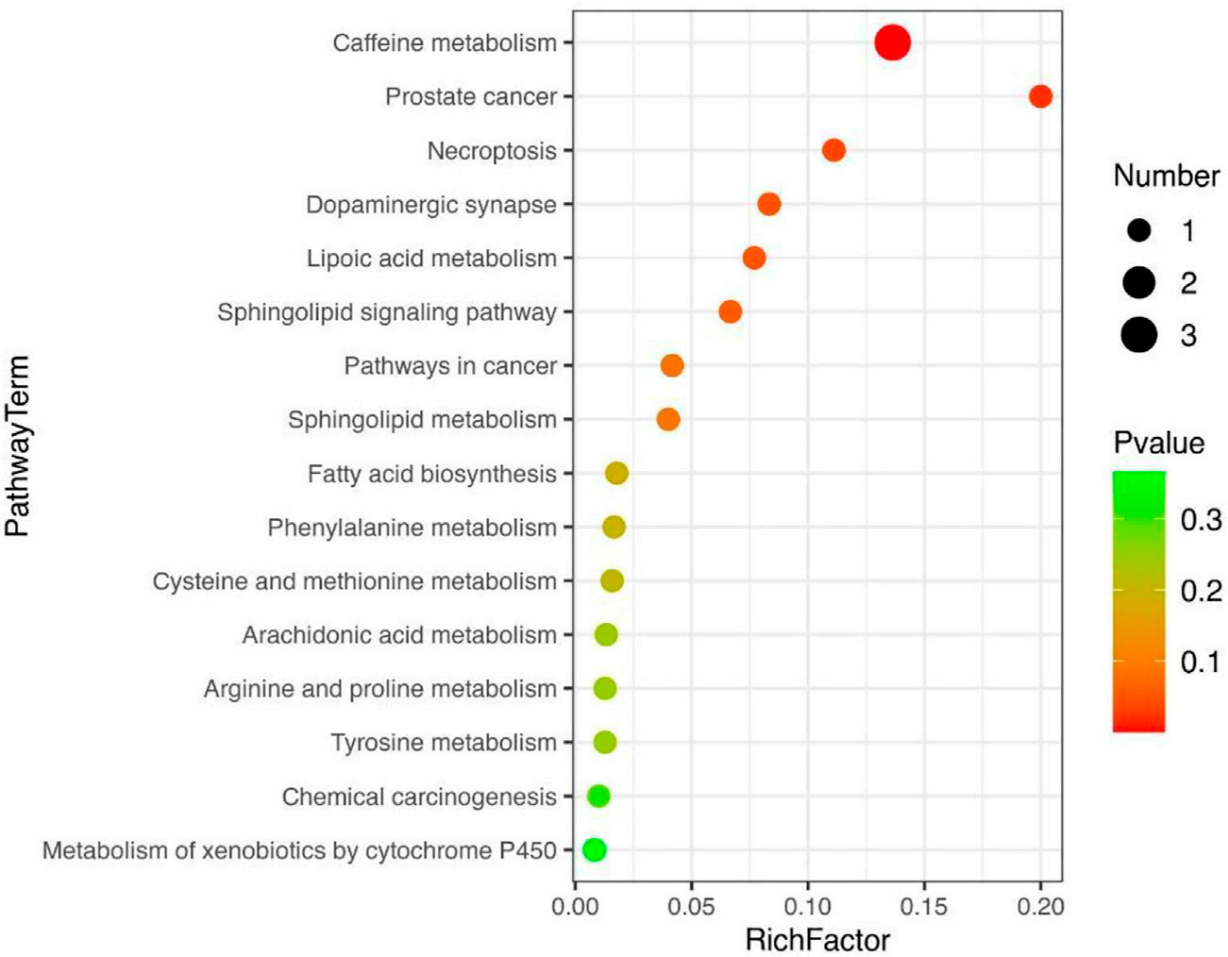
Bubble diagram of KEGG enrichment analysis for metabolomics of clinical trials.

Metabolomic analysis by clinical serum resulted in 2490 metabolites that were obtained, with 1180 in the negative mode and 1310 in the positive mode. The VIP value of the first principal component of the OPLS-DA model was >0.1, and the *p*-value of the *t-*test was <0.05 for differential metabolite identification, and a total of 98 differential metabolites were found (Figure 12). Metabolic pathway enrichment analysis of the differential metabolites based on the KEGG database revealed a total of 16 metabolic pathways (Figure 13). By comparing the metabolomics results of this clinical trial with the prediction results of network pharmacology, only the cancer pathway (hsa05200) was found to be its common pathway among the top 32 signaling pathways predicted by network pharmacology.

The results of extended network pharmacology prediction indicated that the sphingolipid signaling pathway (hsa04071) and prostate cancer pathway (hsa05215) matched the predicted metabolic pathway, indicating that HXQR may be involved in the treatment of CHD through three signaling pathways, including the cancer pathway (hsa05200), sphingolipid signaling pathway (hsa04071), and prostate cancer pathway (hsa05215). The differential metabolites may be N-oleoyl-D-sphingomyelin (d18:1/22:0) (SM, upregulated), 1-methyl-6-phenyl-1h-imidazole[4,5-b]pyridine-2-amine and 1-phenyl-6-phenyl-1H-imidazo[4,5-b]pyridine-2-amine (downregulated).

**FIGURE 14:**
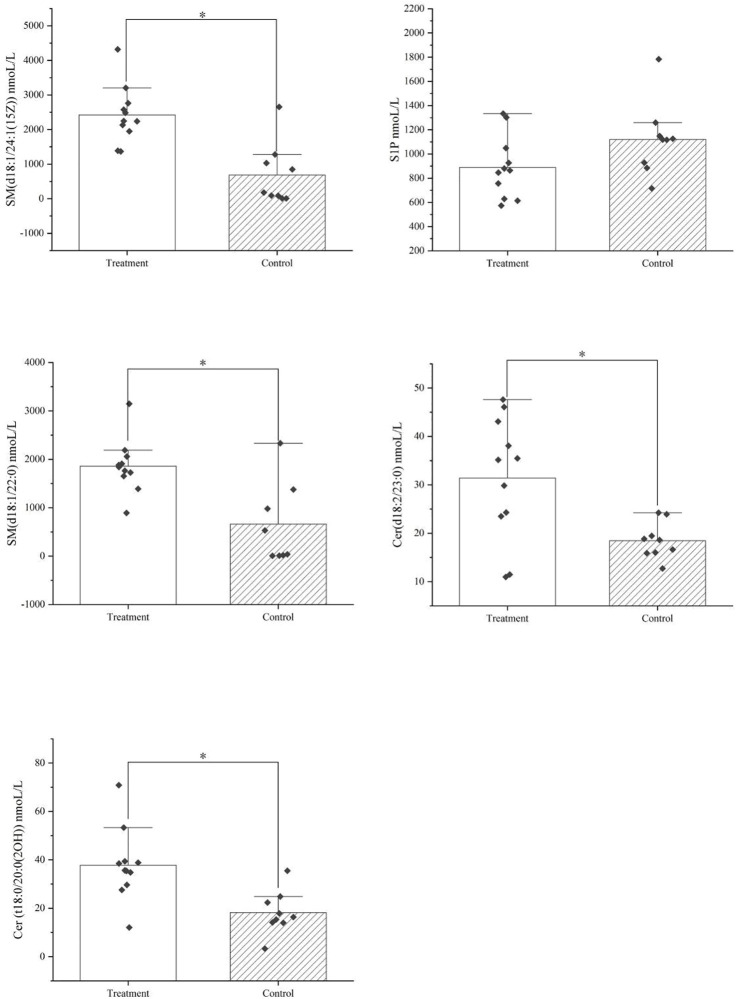
The key metabolites of the sphingolipid signaling pathway (hsa04071) after treatment with HXQR.

The metabolomics results showed that after treatment with HXQR, the key metabolites of the sphingolipid signaling pathway (hsa04071), Cer (d18:2/23:0), and Cer (t18:0/20:0 (2OH)), SM (d18:1/22:0), and SM (d18:1/24:1 (15Z)), were significantly increased (*p* < 0.01), and the level of S1P in serum was decreased (*p* = 0.08) (Figure 14). 1-Methyl-6-phenyl-1H-imidazo[4,5-b]pyridin-2-amine is a key differential metabolite of prostate cancer (hsa05200) and pathways in cancer (hsa05215), which may lead to DNA damage, but the specific cause is not clear and requires in-depth study.

The level of Cer (d18:2/23:0), Cer (t18:0/20:0 (2OH), SM (d18:1/22:0), and SM (d18:1/24:1 (15Z)), were significantly increased after treatment with HXQR (*p* < 0.01), and the level of S1P in serum was decreased after treatment with HXQR (*p* = 0.08).

## Discussion

Modern medical research indicates that the human body is an organism with a complex network. Cardiovascular and cerebrovascular diseases, cancer, tumors and other chronic diseases are not driven by a single cause. Only by analyzing a variety of signal transduction pathways of diseases can achieve a final ideal therapeutic effect. TCM compound preparations are widely used in clinical practice, with significant effect, and play an important role in disease prevention and treatment. However, how to deeply elucidate the complex chemical system and mechanism of action of TCM from the molecular level and establish a complete system including active ingredients and targets has become the focus and difficulty of modernization research of TCM. Network pharmacology provides a new method and reference for the study of TCM, which is consistent with the overall mechanism of action of TCM “multiple components and multiple targets” and has received the attention and favor of the majority of researchers. However, from our group’s analysis of numerous studies related to network pharmacology, we can conclude that the following problems may need to be solved in current network pharmacology research.

It is unreasonable to evaluate chemical compositions with only DL and OB values, and sometimes the conclusions drawn are unscientific or even incorrect. Network pharmacology generally uses OB ≥30% and DL ≥0.18 as conditions to evaluate active ingredients, ignoring some components with unqualified OB values or/and DL values such as tanshinones, ginsenosides, notoginsenosides, puerarin, verbascoside, and echinacoside that play a major role. Therefore, it would be helpful to perform a comprehensive analysis that fully considers the possible active ingredients recorded in the Chinese Pharmacopoeia and authoritative studies while using DL and OB values. In the current study, the project team adopted OB ≥30% and DL ≥0.18, and evaluated the active ingredients in HXQR in combination with the active ingredients indicated in the Chinese Pharmacopoeia.

There is still a space for improvement in the TCMSP and analysis platform. At present, the active ingredients and targets of some commonly used TCMs are missing, resulting in serious biases in the results and conclusions. For example, it is debatable that most of the network pharmacology literature will now conclude that β-sitosterol is the active ingredient. β-Sitosterol is widely presented in nature, and confers a variety of pharmacological effects, but the network pharmacological research literature that has used β-sitosterol as an active ingredient is worth pondering. The TCMSP database currently lacks commonly used active ingredients, and contains information errors and other circumstances that require continuous improvement by researchers for optimal and rational use.

Network pharmacological results need to undergo a series of validations to draw scientific conclusions. In this study, 151 active ingredients and 286 targets of HXQR in the treatment of CHD were obtained by network pharmacology research technology, and 81 common targets were obtained by mapping the active ingredient targets of HXQR with CHD targets. KEGG pathway enrichment analysis showed that 105 pathways were related to them, 32 pathways were obtained by analyzing with *p* < 0.05, and the number of genes was greater than or equal to 10. In terms of compounds, quercetin (MOL000098), puerarin (MOL012297), luteolin (MOL000006), kaempferol (MOL000422), tanshinone iia (MOL007154), and baicalein (MOL002714) were associated with multiple targets and may be the main active components of HXQR decoction in the treatment of CHD. *In vitro* and *in vivo* experiments have also confirmed that quercetin has the effects of antihypertensive, regulating blood lipid, lowering blood pressure, anti-atherosclerosis and inhibiting cardiotoxicity (Papakyriakopoulou et al., 2022). Kaempferol flavonoid compounds have antioxidant and antiplatelet potential and can improve cardiovascular disease by improving oxidative stress (Olas, 2020). Puerarin, as a natural isoflavone, has been developed into a variety of injectable dosage forms for widespread use. It can be used for a variety of diseases such as atherosclerosis, cardiac hypertrophy, heart failure, myocardial infarction and hypertension and other diseases (Zhou et al., 2021). Luteolin has antioxidant and anti-inflammatory effects, and has protective effects in various diseases such as ischemia/reperfusion (I/R) injury, heart failure (HF) and atherosclerosis (AS) and can inhibit apoptosis (Xu et al., 2012; Luo et al., 2017). Tanshinone iia is well studied, also has dosage forms such as injections, has anti-inflammatory and antioxidant activities, and can induce significant cardioprotection by enhancing angiogenesis (Guo et al., 2020). Baicalein can intervene signaling molecules and protect cardiomyocytes from ischemia/hypoxia injury by acting on PI3K/Akt, MAPKs, and NF-κB/p65 signaling pathways (He et al., 2015). The data from the above studies also indicate the intervention effect of the main active ingredients on cardiovascular disease and also provide support for docking.

Metabolomics of clinical trials of HXQR for the treatment of CHD showed that the differential metabolites were identified by VIP value of the first principal component of the OPLS-DA model >0.1 and *p*-value value of *t-*test < 0.05, with only 98 differential metabolites, which were enriched for metabolic pathways and could correspond to 16 pathways. In comparing these 16 clinically proven effective pathways with the network pharmacology prediction results, only three pathways were found to be identical, which may be related to the small sample size in this study. This result demonstrated that HXQR played a role in the treatment of CHD through three signaling pathways: the cancer pathway (hsa05200), the sphingolipid signaling pathway (hsa04071), and the prostate cancer pathway (hsa05215). However, we found that these three signaling pathways only had cancer pathway (hsa05200) in the top 32 predicted pathways, and prostate cancer pathway (hsa05215) and sphingolipid signaling pathway (hsa04071) in the 36th and 60th of the KEGG prediction results of network pharmacology, respectively, which also indicated that the unvalidated network pharmacology results may be severely biased and must be subject to the large sample size and strict scientific validation before arriving at any scientific conclusions.

SM is mainly located on the cell membrane, lipoproteins (especially LDL), and other lipid-rich tissue structures, which is very important for maintaining the micro-control function of the cell membrane structure so that it can regulate the activity of growth factor receptors and extracellular matrix proteins. Its degradation and anabolic intermediates are known as sphingomyelins, which have the effect of regulating cell biological functions (Cirillo et al., 2021). The metabolites of SM include ceramide (Cer) and sphingosine-1-phosphate (S1P), of which Cer is a central molecule in sphingomyelin metabolism, and its biological functions mainly include inducing apoptosis, and the regulation of cell differentiation, cellular immunity, and inflammatory responses. In this process, sphingomyelinase (SMase) is the key enzyme regulating SM metabolism, which can decompose SM to produce Cer and phosphorylcholine. Cer is cleaved to sphingosine (Sph) by ceramidase (CDase), and phosphatidylcholine generates S1P by sphingosine kinase (SphK), which then activates the downstream MAPK, BAX/BCL-2, and PI3K/AKT signaling pathways involved in the regulation of cell proliferation and apoptosis (Nishino et al., 2019; Cirillo et al., 2021; Green et al., 2021). The current study showed that higher plasma levels of Cer-16 and SM-16 were associated with increased risk of heart failure, and higher levels of Cer-22, SM-20, SM-22, and SM-24 with decreased risk of heart failure (Lemaitre et al., 2019; Fretts et al., 2021). Plasma S1P and sphingomyelin levels were significantly negatively correlated with the left ventricular ejection fraction and the severity of dyspnea (Polzin et al., 2017; Deshpande et al., 2018). This also demonstrates the consistency of the clinical trial evaluation with the network pharmacological analysis.

## Conclusion

Through network analysis and metabolomic evaluation, there may be three signaling pathways that involve the Huoxue Qingre decoction in the treatment of CHD: pathways in cancer (hsa05200), sphingolipid signaling pathway (hsa04071), and prostate cancer pathway (hsa05215). Network pharmacology provides a method reference for the study of modernization of TCM. However, it is necessary to recognize the existing problems of network pharmacology and adopt a reasonable method to solve these problems in order to draw possible and correct conclusions.

## Data Availability

The datasets presented in this study can be found in online repositories. The names of the repository/repositories and accession number(s) can be found in the article/Supplementary Material.

## Abbreviations

ADRB2, β-2 adrenergic receptor; AR, androgen receptor; BP, biological process; CC, cellular component; CDase, ceramidase; Cer, ceramide; CHD, Coronary heart disease; DL, drug-like; ESR1, estrogen receptor; GO, Gene Ontology; HXQR, Huoxue Qingre decoction; KCNH2, potassium voltage-gated channel subfamily H member 2; KEGG, Kyoto Encyclopedia of Genes and Genomes; MF, molecular function; NOS2, inducible nitric oxide lyase; NOS3, endothelial nitric oxide synthase; OB, oral bioavailability; PPARG, peroxisome proliferator-activated receptor γ; PPI, protein-protein interaction; PTGS2, prostaglandin G/H synthase 2; S1P, sphingosine-1-phosphate; SCN5A, sodium channel protein type 5 subunit α; SLC6A4, sodium-dependent serotonin transporter; SM, N-oleoyl-D-sphingomyelin; SMase, sphingomyelinase; Sph, sphingosine; SphK, sphingosine kinase; TCM, traditional Chinese medicine; TCMSP, Traditional Chinese Medicine Systems Pharmacology Database.
