# Supplementary material for: Huoxue Qingre decoction used for treatment of coronary heart disease network analysis and metabolomic evaluation
Source: Front Pharmacol. 2022 Oct 20;13:1025540. doi: 10.3389/fphar.2022.1025540 (PMC9631828; doi:10.3389/fphar.2022.1025540)
Supplement: Supplementary file 1 [file DataSheet1.docx]

**The Attachment Material**

**Huoxue Qingre Decoction used for Treatment of Coronary Heart Disease Network Analysis and Metabolomic Evaluation**

Yu-Qing Tan, Min Jin, Xuan-Hui He*, Heng-Wen Chen*

*Department of Pharmacy, Guang’anmen Hospital, China Academy of Chinese Medical Sciences, Beijing 100053, China*

Yu-Qing Tan and Min Jin contributed equally to this work.

* Correspondence: Heng-Wen Chen, chenhengwen@163.com; Xuan-Hui He, hexuanzi1646@sina.com

**Design of the clinical experiments**

**1 Selection of study subjects**

1.1 Case Source

The recruitment of subjects started from December 2017. All subjects who met the inclusion and exclusion criteria could be enrolled only after voluntarily signing the informed consent form. It was planned to enroll 32 subjects (test group: placebo group = 1:1).

1.2 Diagnostic criteria

1.2.1 Diagnostic criteria of western medicine

The diagnostic criteria for borderline coronary lesions refer to the “2011 ACCF/AHA/SCAI Guidelines for Percutaneous Coronary Intervention”, which are specifically defined as a coronary artery diameter stenosis of 30%-70%. The diagnostic criteria for stable angina pectoris of coronary heart disease refer to the “Guidelines for the Diagnosis and Treatment of Patients with Stable Ischemic Heart Disease (2012 Edition)”. The diagnostic criteria for unstable angina pectoris of coronary heart disease refer to the “Guidelines for the Management of Patients with Non-ST Segment Elevation Acute Coronary Syndrome (2014 Edition)”.

1.2.2 Diagnostic criteria for TCM syndromes

The diagnostic criteria for angina pectoris of coronary heart disease with phlegm-blood stasis and heat syndrome refer to the “Diagnostic Criteria for Syndrome Factors of Angina Pectoris of Coronary Heart Disease” issued by the Cardiovascular Branch of the Chinese Society of Traditional Chinese Medicine.

1.3 Inclusion Criteria

(1) Written informed consent voluntarily signed by the patient or agent; (2) Patients aged 30 to 75 years; (3) Identify at least one target plaque in the 30% to 70% diameter stenosis range by coronary CTA, the nature of the target plaque is non-calcified plaque or mixed plaque, and the target plaque is located in the left anterior descending branch (LAD), circumflex artery (CX), or right coronary artery (RCA); (4) The diagnosis of TCM syndrome belongs to the phlegm-blood stasis and heat.

1.4 Exclusion Criteria

1.4.1 Chest pain caused by severe left main coronary artery disease, severe valvular heart disease (aortic stenosis), cervical spondylosis, biliary heart disease, gastric and esophageal reflux, and other non-coronary heart diseases;

1.4.2 Patients with poorly controlled hypertension, severe cardiopulmonary dysfunction (LVEF% < 35%), and severe arrhythmia;

1.4.3 Patients with severe primary diseases such as visceral system, liver function ALT or AST values > 1.5 times the upper limit of normal value, abnormal renal function, or insulin-dependent diabetes;

1.4.4 Coronary CTA cannot measure the indicators required for this trial due to severe tortuosity of the vessel where the target plaque is located or other anatomical reasons;

1.4.5 Patients with target plaque underwent revascularization including PCI or coronary artery bypass grafting (CABG);

1.4.6 Patients with depression or anxiety;

1.4.7 Stroke within the past 6 months;

1.4.8 Hyperthyroidism (TSH value > 1.5 times the upper limit of normal);

1.4.9 Patients with malignant tumors;

1.4.10 Pregnant or lactating women;

1.4.11 Hormonal drugs (excluding topical, inhaled, or nasal steroids) are required due to chronic diseases;

1.4.12 Allergic constitution and allergy to multiple drugs, such as iodine allergy;

1.4.13 Patients with poor compliance and low possibility of follow-up;

1.4.14 Those who participated in other clinical trials within the past 1 month.

1.5 Criteria for eliminated and dropped cases

1.5.1 Cases that violated the inclusion criteria;

1.5.2 Cases without medication after inclusion;

1.5.3 Myocardial infarction in target plaque and treated with PCI or CABG during intervention;

1.5.4 Failing to complete relevant examinations according to the test requirements.

1.6 Criteria for termination

1.6.1 In case of serious safety problems during the trial, the trial should be discontinued in a timely manner.

1.6.2 The drug treatment was found to be ineffective in the trial.

**2 Study methods**

2.1 Research Design

A randomized, double-blind, placebo-controlled clinical trial method was used.

2.2 Randomization and Blinding

SAS statistical software was used to generate the randomization code according to the allocation ratio of 1:1 between the experimental group and the control group, and the patients were numbered and allocation concealment was performed by sealing the code in radiopaque paper envelopes. Randomization and allocation concealment were performed by a third party who did not participate in the trial. The granules of HXQR and placebo were provided by Sichuan Xinlvse Pharmaceutical Technology Development Co., Ltd. They have the same appearance, color, and smell, have identical outer packaging, and cannot be distinguished by researchers and subjects to ensure that blinding is performed. The investigator should distribute the drugs strictly according to the order of each visit and the drug number, do not select the drugs, and the number remains stable throughout the trial. Unblinding after the end of the study.

2.3 Treatment method

The subjects in the experimental group were given HXQR granules (7.72 g/bag) on the basis of conventional western medicine treatment (antiplatelets, statins, nitrates, etc.). Medication methods: oral, 1 bag each time, twice a day, hot water rinse, intervention time of 6 months.

The subjects in the control group were given HXQR placebo granules (7.72 g/bag) on the basis of conventional western medicine treatment (antiplatelets, statins, nitrates, etc.). The placebo composition was: 10% HXQR formula ingredients, soluble starch, mixed colorants, and bitter tastants. Medication methods: oral, 1 bag each time, twice a day, hot water rinse, intervention time of 6 months.

2.4 Outcome measures

2.4.1 General data (screening period 1 week before treatment)

2.4.1.1 Demographic data: gender, age, height, weight, etc.

2.4.1.2 General clinical data: medical history, smoking and alcohol history, angina pectoris type, past medical history, family history and combined use of drugs, etc.

2.4.2 Efficacy indicators

2.4.2.1 Target plaque efficacy

2.4.2.1.1 Target plaque efficacy indicators: the degree of luminal area stenosis where the target plaque is located, the degree of luminal diameter stenosis where the target plaque is located, the target plaque volume, the average CT value of the target plaque, and the target plaque calcification score (before treatment and after 6 months of treatment).

2.4.2.1.2 Screening and analysis of target plaques

The target plaques to be detected are non-calcified plaques or mixed plaques with diameter stenosis between 30% and 70%, and the target plaques are located in the LAD, CX or RCA. Measuring the distance from the target plaque to the coronary ostium as a benchmark index was used to locate the location of the target plaque to ensure the accuracy of measuring the target plaque again after treatment. The same workstation was used by two professional fellows in the radiology department of Guang'anmen Hospital (Syngo.via VB10B) and quantitative CT was independently used to quantitatively measure target plaque efficacy indicators before and after treatment.

2.4.2.2 Efficacy indicators of blood lipid: total cholesterol (TC), triglyceride (TG), low-density lipoprotein (LDL), high-density lipoprotein (HDL), and very low-density lipoprotein (VLDL) (before treatment and after the 3rd and 6th month of treatment).

2.4.2.3 Inflammatory indicators: High-sensitivity C-reactive protein (hs-CRP) (before treatment and after the 3rd and 6th month of treatment).

2.4.2.4 TCM efficacy evaluation of angina pectoris of coronary heart disease (before treatment and after 1st, 2nd, 3rd, 4th, 5th, and 6th months of treatment).

2.4.2.5 Efficacy evaluation of TCM syndrome score (before treatment and after 1st, 2nd, 3rd, 4th, 5th, and 6th months of treatment).

2.4.2.6 Seattle Angina Questionnaire (SAQ) (before treatment and after 1st, 2nd, 3rd, 4th, 5th, and 6th months of treatment). SAQ includes 19 questions on 5 major items, which are independently completed by the subject after being explained to the subject by the investigator. Nineteen questions of the five major items of the SAQ were scored separately. The standard score of each item = (the actual score of each item - the lowest score of the item)/(the highest score of the item - the lowest score of the item) × 100%. Higher scores represent higher quality of life status for patients.

2.4.3 Safety indicators

2.4.3.1 Vital signs: such as body temperature, heart rate, respiration, blood pressure, etc. (before treatment and after 1st, 2nd, 3rd, 4th, 5th, and 6th months of treatment).

2.4.3.2 Blood routine, urine routine and stool routine (before treatment and after the 3rd and 6th month of treatment).

2.4.3.3 Liver and kidney function (before treatment and after the 3rd and 6th month of treatment).

2.4.3.4 Electrocardiogram (before treatment and after the 6th month of treatment).

2.4.3.5 Adverse events (documented in detail on the adverse event report form at any time).

2.5 Quality control of data

We will take effective measures to ensure the accuracy, completeness, and authenticity of the information. Firstly, all investigators will be trained on patient screening, data completion, adverse event reporting, and other matters. Secondly, the trial inspectors will regularly check the electronic database and ensure that the trial is in strict compliance with the protocol. Thirdly, the data coordinating center will be responsible for data validation. Fourthly, investigators should take measures to control dropout rates within 15%.

2.6 Statistical analysis

In this study, SPSS Statistics 20.0 was used for statistical analysis of data. Measurement data conforming to normal distribution were expressed as mean ± standard deviation, and t-test was used; measurement data not conforming to the normal distribution were presented as median and quartiles, and wilcoxon rank sum test was used. Enumeration data were expressed as number and percentage, and χ^２^test was used. All statistical tests were two-sided, and *P* < 0.05 was considered statistically significant.

2.7 Ethical review and clinical trial registration

This trial conforms to the ethical requirements, and has been approved by the Ethics Committee of Guang'anmen Hospital, China Academy of Chinese Medical Sciences (Approval No.: 2017-083-KY-01), and has completed the Chinese clinical trial registration (Registration No.: ChiCTR-IOR-17013189).

**Metabolomics detection methods and result analysis process are as follows**

1 Reagents and instruments

1.1 Reagents

Methanol, formic acid, water and acetonitrile were purchased from CNW Technologies GmbH (GER), L-2-Chlorophenylalanine was purchased from Shanghai HC Biotech Co., Ltd. (CHN), and LysoPC (17:0) was purchased from Avanti Acquisition Corp. (USA). All chemicals and solvents were analytical grade or chromatographic grade.

1.2 Instruments

Ultrasonic Cleaner (SB-5200DT, Ningbo Scientz Biotechnology Co. Ltd., CHN), Vortex (TYXH-I, Shanghai Hannuo Instrument Co. Ltd., CHN), Desktop high speed freezing centrifuge (TGL-16MS, Shanghai luxianyi centrifuge instrument Co. Ltd., CHN), High resolution mass spectrometer (AB Triple TOF 5600, AB Sciex Pte. Ltd., CHN), High performance liquid chromatography (ACQUITY UPLC, Waters Corporation, USA), chromatographic column (ACQUITY UPLC BEH C18 (100*2.1 mm,1.7 μm), Waters Corporation, USA).

2 Methods

2.1 Preprocessing

The samples stored at -80°C were taken out, thawed at room temperature, 100 μL of serum was pipetted, and the internal standard (L-2-chlorophenylalanine, 0.3 mg/mL; Lyso PC17:0, 0.01 mg/mL, both methanol configurations) was added 10 μL each, vortexed for 10 s; added 300 μL of protein precipitant methanol-acetonitrile (V:V=2:1), vortexed for 1 min; ultrasonically extracted in ice-water bath for 10 min; standing at -20°C for 30 min; Centrifuge for 10 min (13000 rpm, 4°C), evaporated 300 μL of the supernatant to dryness, then reconstituted with 200 μL of methanol-water (V: V = 1:4), vortexed for 30 s, sonicated for 2 min. Centrifuge for 10 min (13000 rpm, 4°C), draw 150 μL of the supernatant with a syringe, filter it with a 0.22 μm organic-phase pinhole filter, transfer it to an LC injection vial, and store it at -80 °C until LC - MS analysis.

Quality control samples (QCs) were prepared by mixing equal volumes of extracts from all samples, and each QC volume was the same as the sample. All extraction reagents were precooled at -20 ° C prior to use.

2.2 Liquid Chromatography-Mass Spectrometry Conditions

Chromatographic conditions: chromatographic column ACQUITY UPLC BEH C18 (100 mm×2.1 mm, 1.7 μm); column temperature 45°C; mobile phase: (A) water (containing 0.1% formic acid), (B) acetonitrile/methanol (2/3) (v/v) (containing 0.1% formic acid); flow rate: 0.4 mL/min; injection volume: 5 μL; elution conditions: A% (95%–0) gradient elution. Mass spectrometric conditions: electrospray ionization (ESI) ion source, the positive and negative ion scanning modes were used for sample mass spectrometric signal acquisition.

3 Data analysis

3.1 Base Peak Chromatogram

Base Peak Chromatogram (BPC) is a profile obtained by continuously depicting the intensity of the most intense ion in the mass spectrum at each time point. Figures 1-4 are BPCs for positive and negative ion mode.

Figure 1 Positive ion BPC of HXQR group

Figure 2 Positive ion BPC of Placebo group

Figure 3 Negative ion BPC of HXQR Group

Figure 4 Negative ion BPC of Placebo group

3.2 Quality control samples

QC were used to equilibrate the "chromatography-mass spectrometry" system prior to sample testing and to evaluate the stability of the mass spectrometry system during sample testing. The PCA model plot (Figure 5), obtained by 7-fold cross-validation, showed that the QC clustered closely together, indicating that this experiment was stable and reproducible.


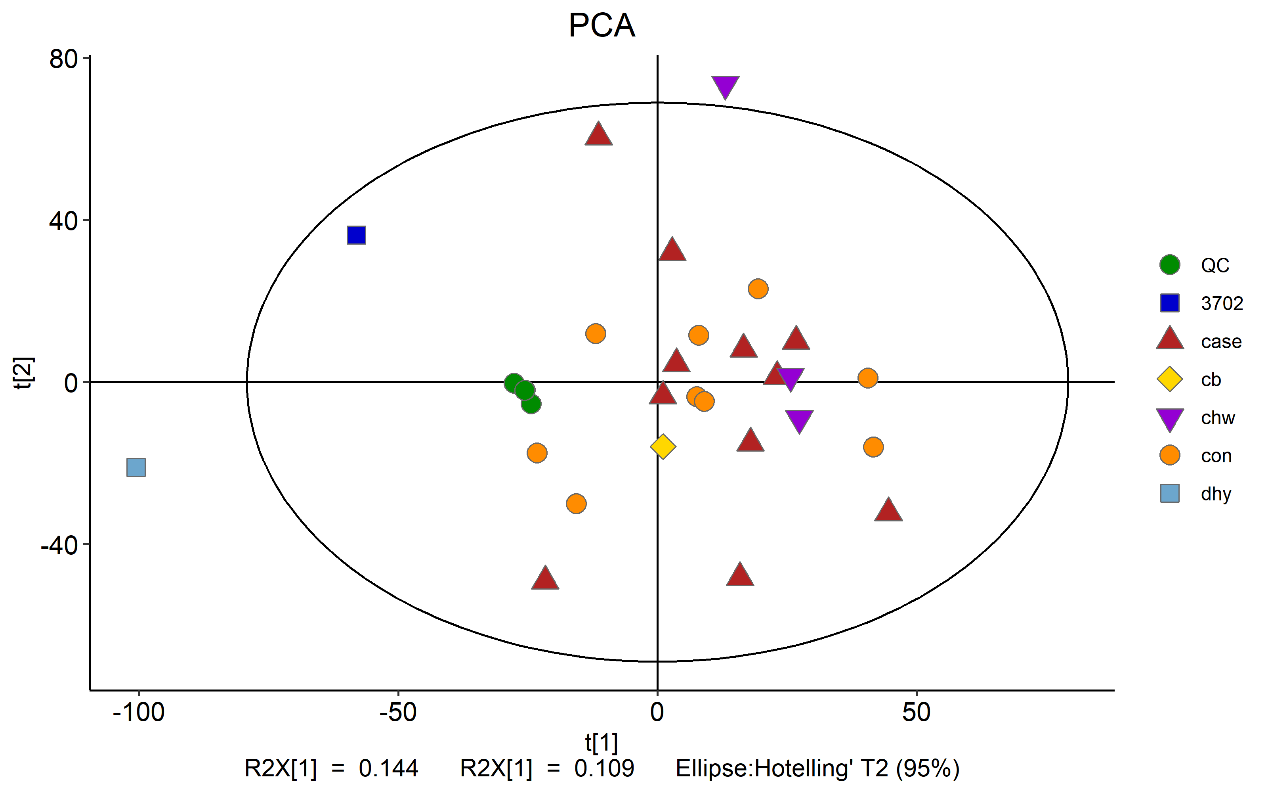


Figure 5 PCA score map of all samples

Case, HXQR group; con, placebo control group.

Boxplot was performed on the metabolite intensities of QC as shown in Figure 6, where Y coordinate is the mass spectral intensity log10 value.


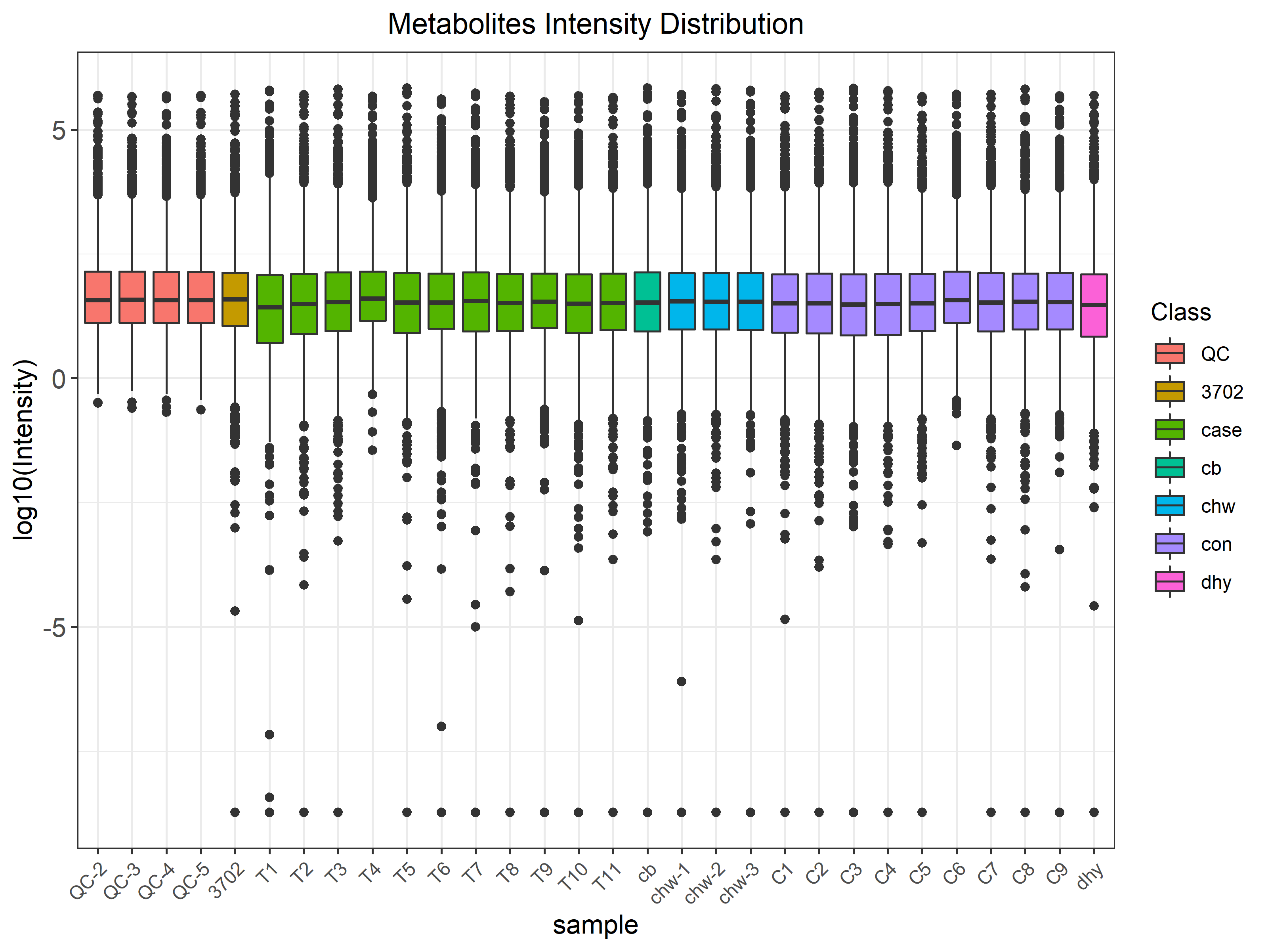


Figure 6 Boxplot of metabolite intensity of all samples

Case, HXQR group; con, placebo control group.

In order to show more intuitively the relationship between QC and other samples and the stability between QC, we performed Hierarchical Clustering for all metabolite expression levels, as shown in Figure 7.


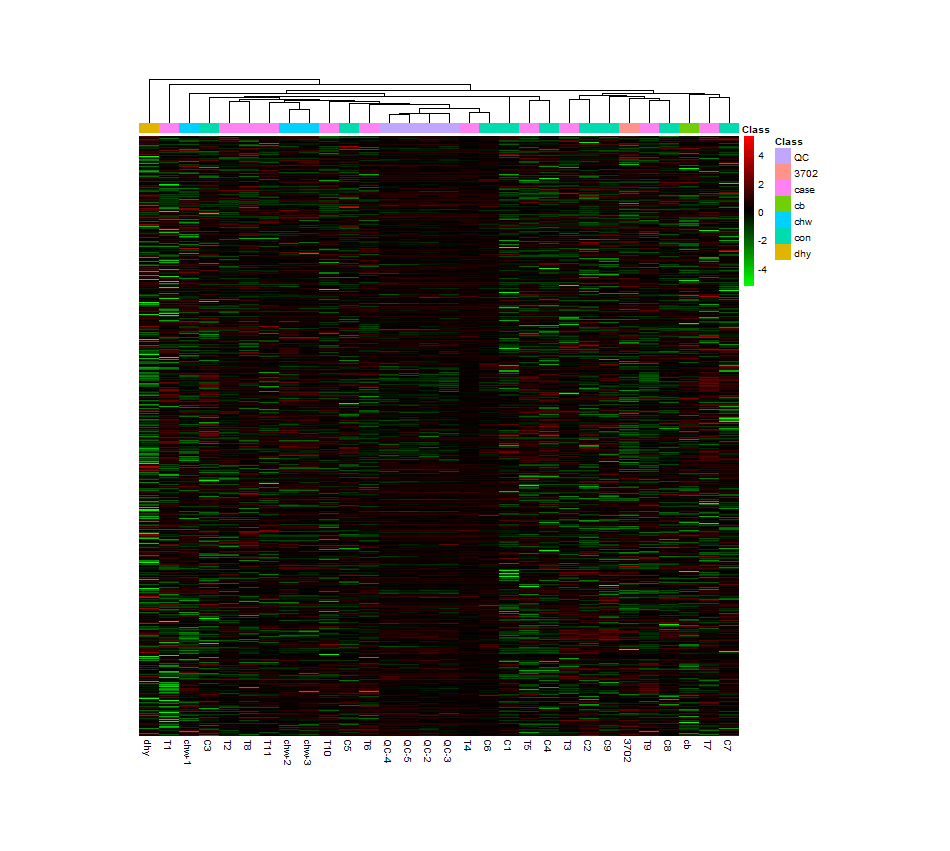


Figure 7 Cluster heatmap of all samples

Case, HXQR group; con, placebo control group.

3.3 Data preprocessing

For the extracted data, ion peaks with missing values (0 value) > 50% were removed, and 0 values were replaced with half of the minimum value, and qualitatively obtained compounds were screened according to the compound qualitative result score, with a screening criterion of 30 points (out of 60 points), and less than 30 points were considered as inaccurate qualitative results and removed. Finally, the positive and negative ion data were combined into a data matrix table (Figure 8), which contained all the information extracted from the original data that could be used for analysis, and subsequent analysis was based on this.


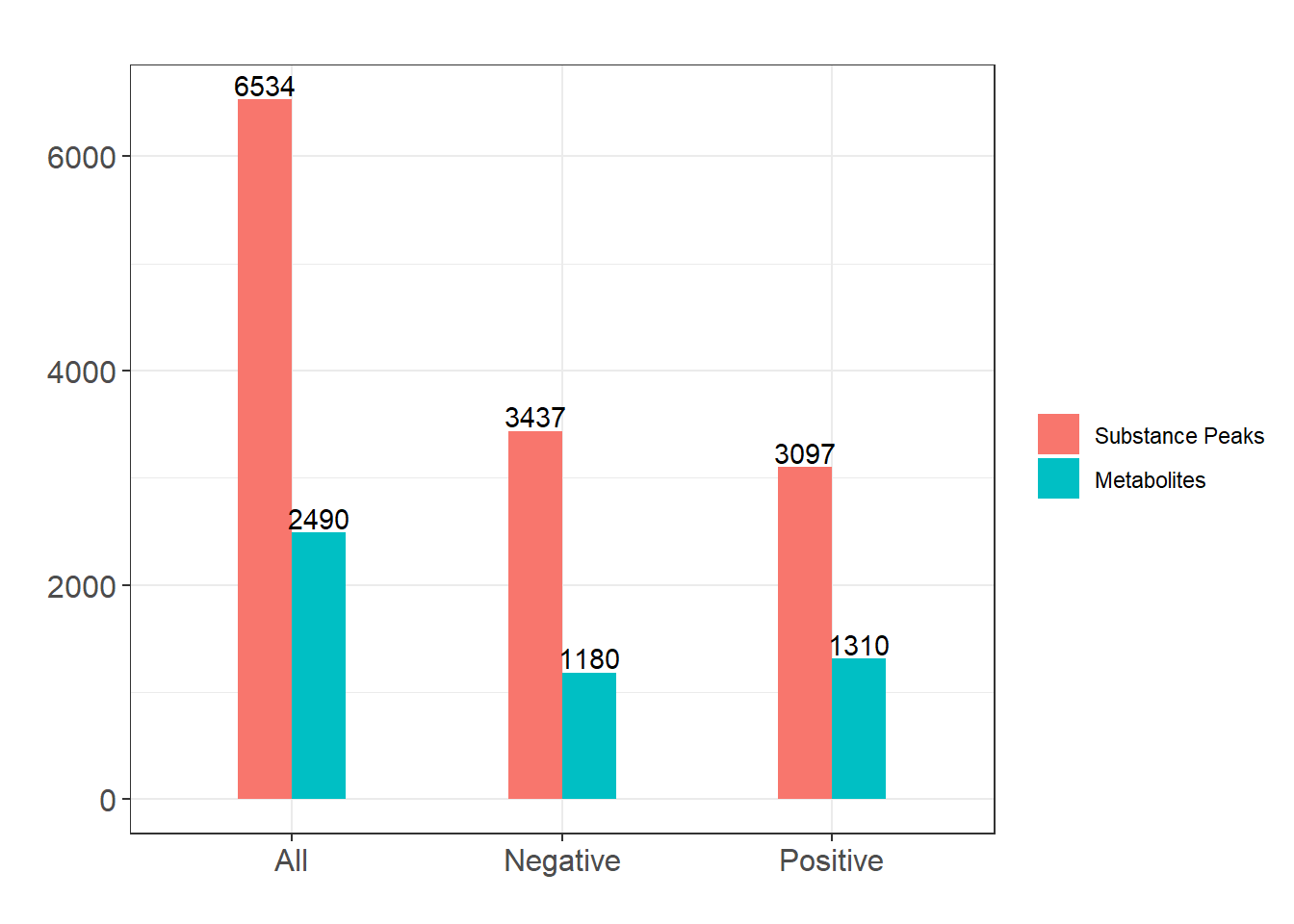


Figure 8 Statistical Plots of Substance Peaks and Metabolites

3.4 Data Analysis

3.4.1 Multivariate statistical analysis

Multivariate statistical analysis will first use unsupervised principal component analysis (PCA) to observe the overall distribution between each sample and the stability of the entire analysis process, and then use supervised partial least squares analysis (PLS-DA) and orthogonal partial least squares analysis (OPLS-DA) to distinguish the overall differences in metabolic profiles between the groups and find differential metabolites between groups. The case/con group was used as an example to perform a comparative analysis of the two groups of samples. Two hundred response ranking tests were performed for PCA, PLS-DA, OPLS-DA analysis, and OPLS-DA model (Figure 9-12). It was found that all R 2 and Q 2 values calculated by random ranking were smaller than the original values, and there was a negative intercept between the Q 2 regression line and the Y axis, indicating that the model was effective without overfitting and could be used to screen metabolite components that resulted in differences between the two groups of samples.


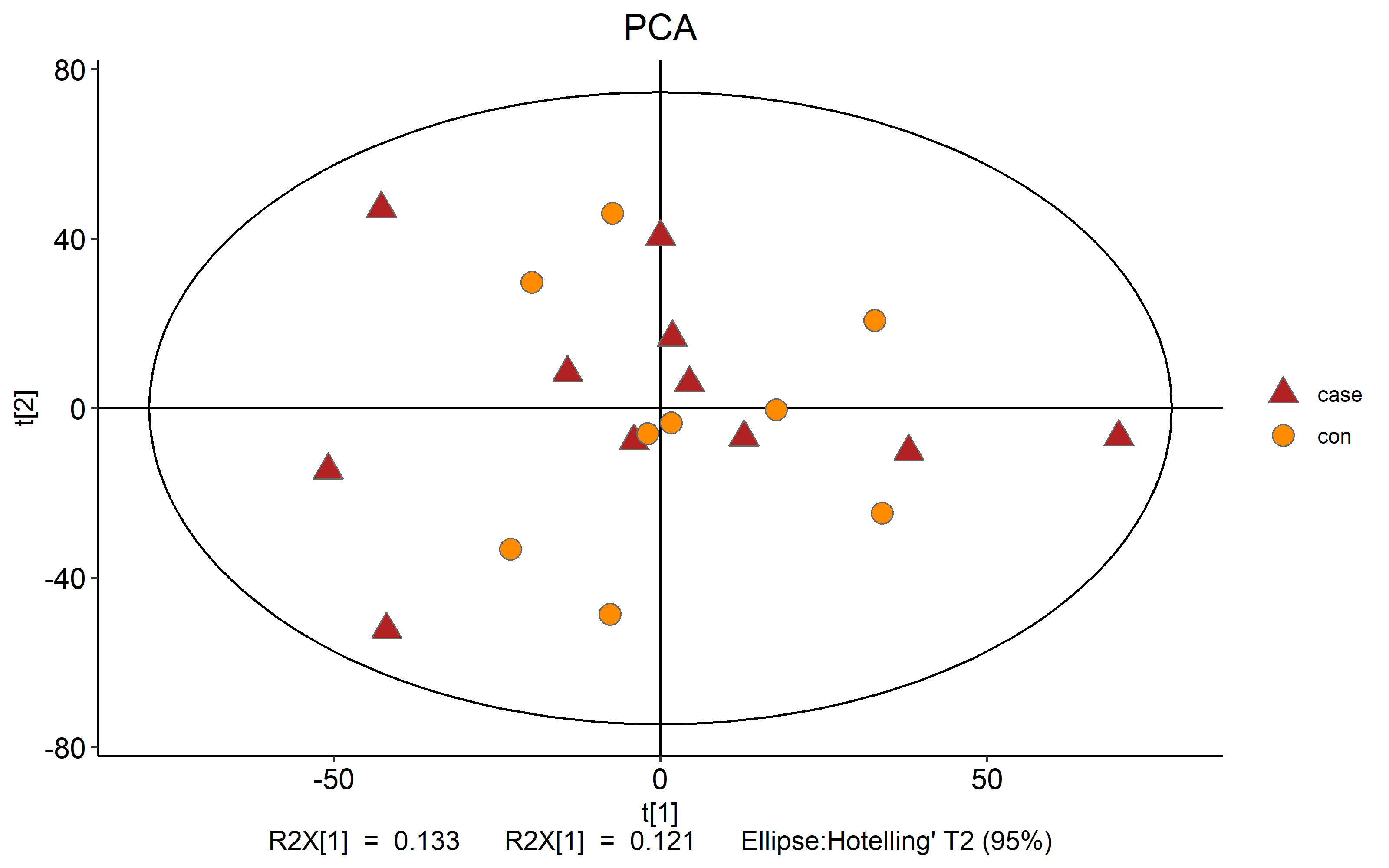


Figure 9 PCA Plot for Case/con Group

Case, HXQR group; con, placebo control group.


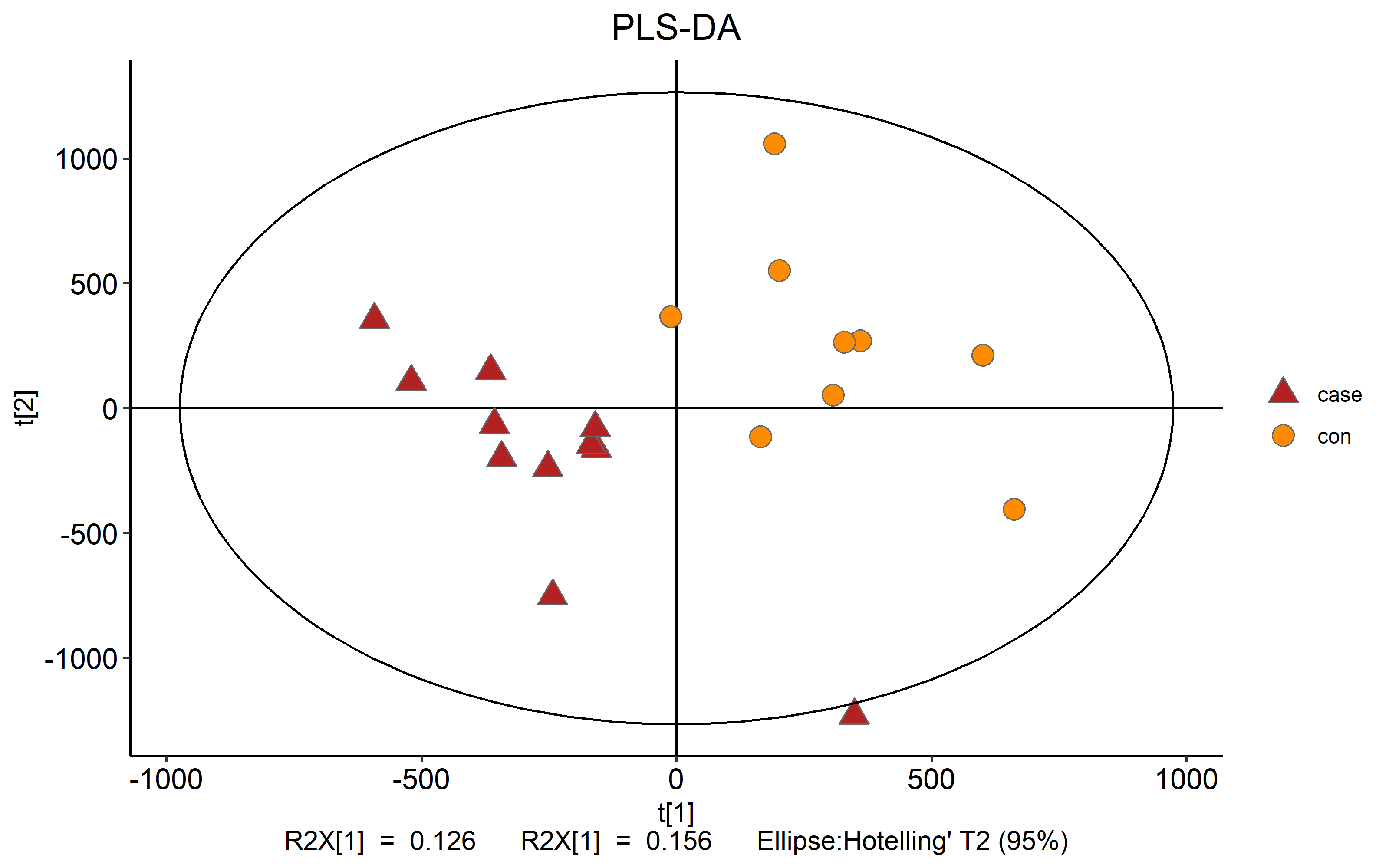


Figure 10 PLS-DA plot for case/con group

Case, HXQR group; con, placebo control group.


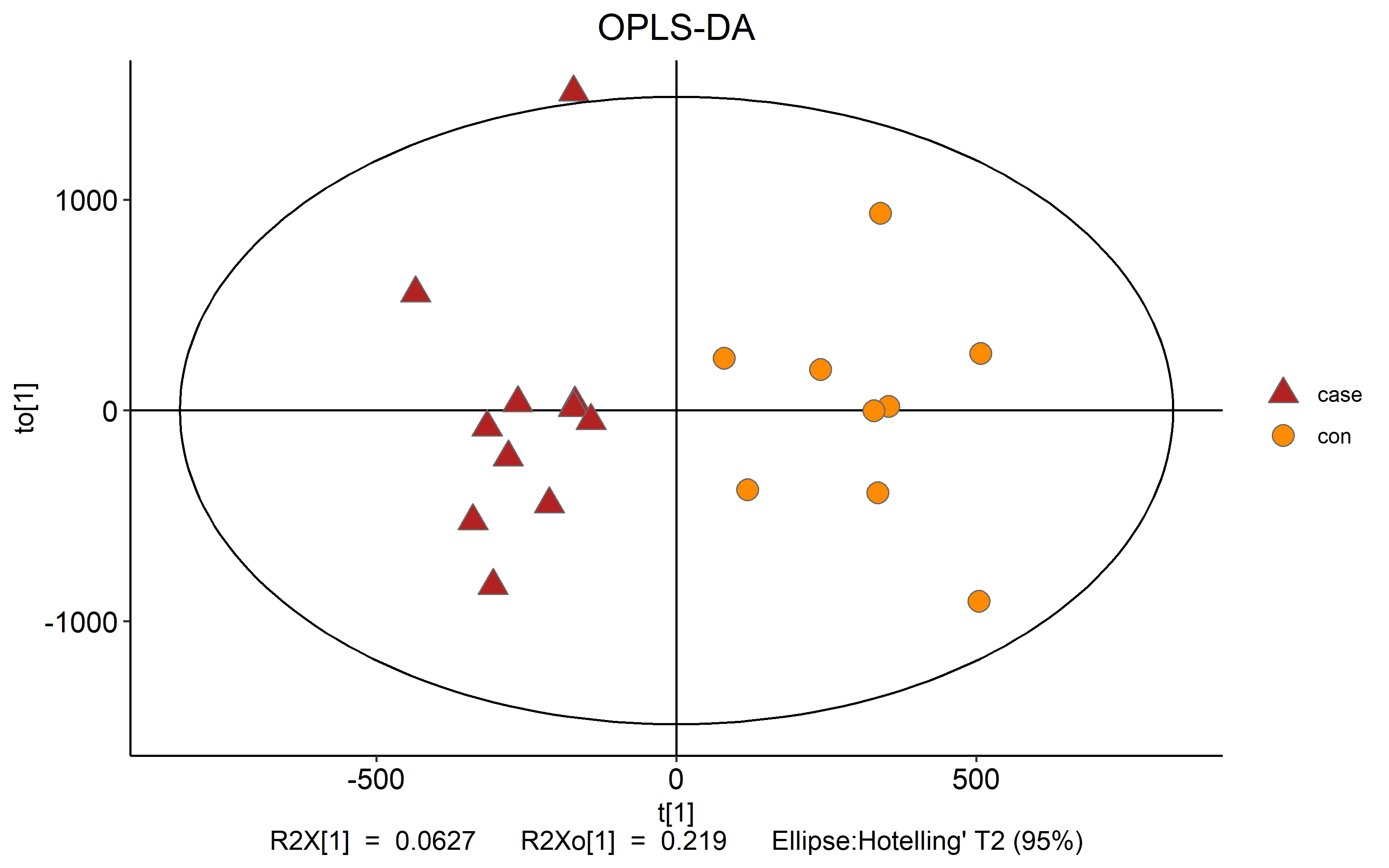


Figure 11 OPLS-DA plot for case/con group

Case, HXQR group; con, placebo control group.


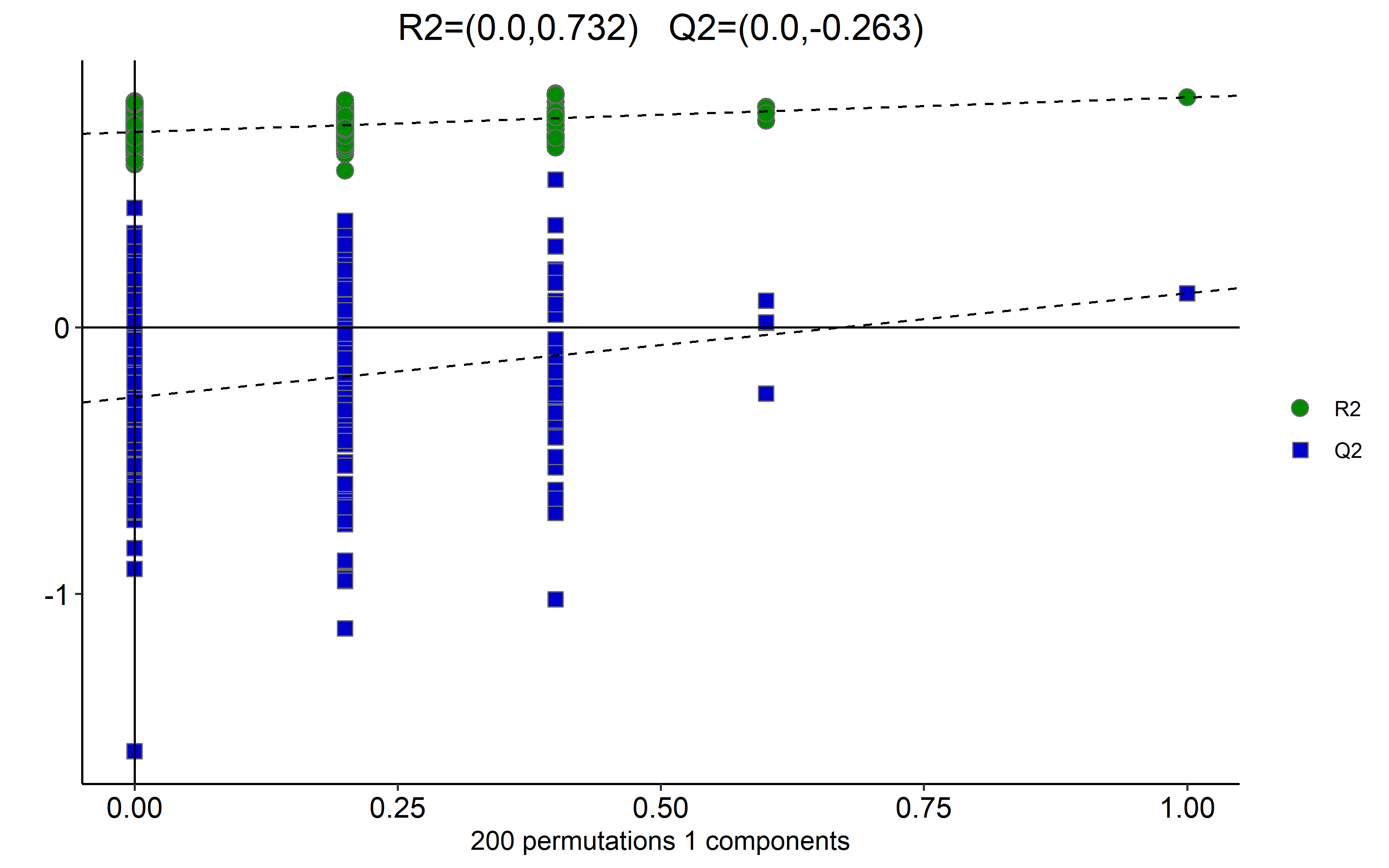


Figure 12 Permutation plot for case/con group

Case, HXQR group; con, placebo control group.

3.4.2 Univariate Statistical Analysis

Volcano plots can be used to visualize the p-values and Fold change values, which facilitate the screening of differential metabolites, as shown in Figure 13. Among them, the red origin represents the differential metabolites that are significantly up-regulated in the experimental group, the blue origin represents the differential metabolites that are significantly down-regulated, and the gray dot represents the insignificant differential metabolites.


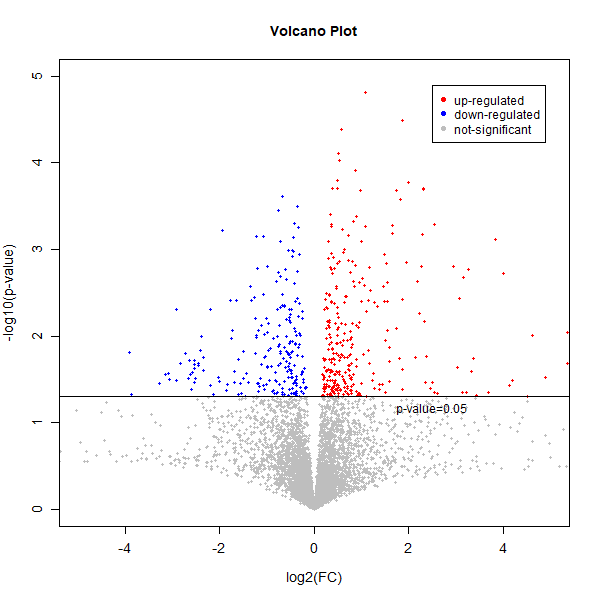


Figure 13 Volcano plot of case/con group

Case, HXQR group; con, placebo control group.
